# Design and Synthesis of New 5-aryl-4-Arylethynyl-1*H*-1,2,3-triazoles with Valuable Photophysical and Biological Properties

**DOI:** 10.3390/molecules26092801

**Published:** 2021-05-10

**Authors:** Mariia M. Efremova, Anastasia I. Govdi, Valeria V. Frolova, Andrey M. Rumyantsev, Irina A. Balova

**Affiliations:** 1 Institute of Chemistry, Saint Petersburg State University (SPbU), Universitetskaya nab. 7/9, 199034 Saint Petersburg, Russia; m.efremova@2012.spbu.ru; 2 Department of Pharmaceutical Chemistry, Saint Petersburg State Chemical Pharmaceutical University (SPCPU), 14A Professor Popov Str., 197376 Saint Petersburg, Russia; zhilyaeva.valeriya@pharminnotech.com; 3 Department of Genetics and Biotechnology, Saint Petersburg State University (SPbU), Universitetskaya nab. 7/9, 199034 Saint Petersburg, Russia; rumyantsev-am@mail.ru

**Keywords:** 1,2,3-triazoles, 1,3-diynes, azide–alkyne cycloaddition, Suzuki-Miyaura cross-coupling, fluorescence, solvatochromism, antimicrobial activity, cytotoxity

## Abstract

Cu-catalyzed 1,3-dipolar cycloaddition of methyl 2-azidoacetate to iodobuta-1,3-diynes and subsequent Suzuki-Miyaura cross-coupling were used to synthesize new triazoles derivatives: 5-aryl-4-arylethynyl-1*H*-1,2,3-triazoles. Investigation of their optical properties by using UV absorption and fluorescence emission spectroscopies revealed that all molecules possess fluorescence properties with the values of the Stokes shift more than 100 nm. The photophysical behavior of the two most promising triazoles in polar and non-polar solvents was also studied.

## 1. Introduction

In recent years, 1*H*-1,2,3-Triazoles are of increasing interest to researchers as one of the most promising classes of heterocyclic compounds for different purposes of bioconjugation [1] design of new catalysts [2], supramolecular ensembles [3,4], and polymeric materials [5]. 1,2,3-Triazoles are widely synthetically available compounds due to the development of simple and effective methods of their preparation on the base of “click” reaction of Cu-catalyzed azide–alkyne cycloaddition (CuAAC) [6,7,8,9,10]. Due to the wide range of available derivatives and the accessibility of structural modifications by a variety of pharmacophore fragments 1,2,3-triazoles attract a special interest in the field of drug design [11,12,13,14]. The biological properties of triazole derivatives are very diverse. Currently, among the most widely studied types of activity are: antibacterial [14,15], antiviral [16], and anticancer [17,18].

Today, a new field of application of triazoles is rapidly developing—using as dyes and fluorophores [19]. For example, CuAAC can be used in the design of push–pull dyes for the formation of triazole linker to connect electron-donating and electron-withdrawing parts of the molecule [20,21,22], in particular, for the synthesis of dyes possessing luminescent properties [23]. It has been shown previously that the luminescent parameters, such as absorption minimum and maximum, Stokes sifts, and quantum yields for triazole derivatives are strongly dependent on the mutual arrangement of substituents in the triazole ring [24,25,26]. For some of the compounds, the dependence of fluorescent properties on the solvent was demonstrated [27,28,29]. However, a limitation of the classical CuAAC method is that the necessity to use terminal acetylenes leads to the possibility of changing substituents only in the first and fourth positions of the triazole ring. The substituents in these positions are not in direct π-conjugation to each other; however, formal cross-conjugation through the triazole system can be recognized for them. Also, triazole derivatives can be used as chemosensors for metal cations due to the combination of photophysical properties with an ability to form complexes due to the presence of lone electron pairs at nitrogen atoms [30,31]. At the same time, 4-ethynyl-1*H*-1,2,3-triazole moiety was not previously used as a linker for fluorophores. 

5-Iodo-1*H*-1,2,3-triazoles are one of the most promising classes of triazole derivatives due to the possibility of caring out in a wide range of C-I functionalization reactions [32]. These compounds are also easily available due to CuAAC, then the 1-idoacetylenes are used instead of terminal acetylenes [33,34]. Earlier, the possibility to involve 1-iodobuta-1,3-diynes in CuAAC and modification of obtained 4-ethynyl-5-iodo-1,2,3-iodotriazoles in cross-coupling reactions were shown by our research group [35].

Herein we report the design and synthesis of new 5-aryl-4-arylethynyl-1*H*-1,2,3-triazoles with valuable photophysical and biological properties, using the synthetic approach included CuAAC of azides to iodobuta-1,3-diynes and subsequent Suzuki-Miyaura cross-coupling.

## 2. Results and Discussion

### 2.1. Synthesis and Structural Characterization

**Scheme 1:**
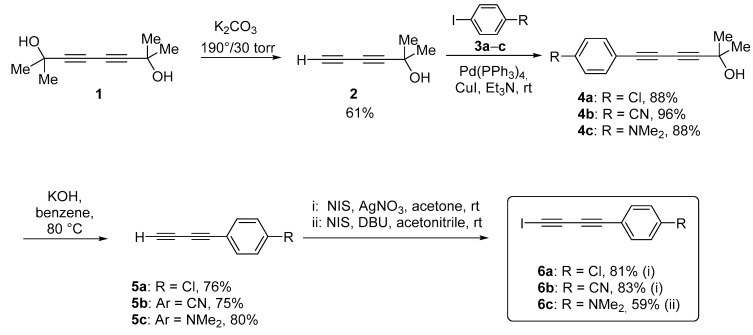
Synthesis of iodobuta-1,3-diynes **6a**–**c**.

For the synthesis of starting iodobuta-1,3-diynes, the four-stage synthetic route was used (Scheme 1). The effectiveness of this approach was shown previously in our research group [35]. At the first stage, 2,7-dimethylocta-3,5-diyne-2,7-diol **1** was subjected to retro-Favorskii reaction by the heating with K_2_CO_3_ [36,37] to synthesize 2-methylhexa-3,5-diyn-2-ol **2,** which was subjected to Sonogashira coupling with iodoarenes **3a**–**c** [38]. Obtained compounds **4a**–**c** were involved in retro-Favorskii reaction again using KOH. Depending on the substituents in the aryl fragment, one of two methodologies of iodination was used: for electron-withdrawing substituted acetylenes **5a** and **5b** NIS in the presence AgNO_3_ of (i) or DBU (ii) for diacetylene **5c** with dimethylamino group. 

**Scheme 2:**
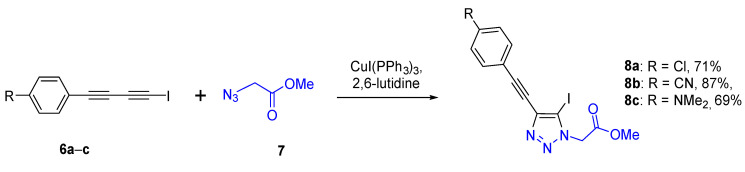
Cycloaddition of azide **7** to iodobuta-1,3-diynes **6a**–**c**.

Convenient and eco-friendly solvent-free methodology using CuI(PPh_3_)_3_ in the presence of 2,6-lutidine as catalytic system was used for the CuAAC reaction of iodobuta-1,3-diynes **6a**–**c** with methyl 2-azidoacetate **7** [39]. Recently, the efficiency of this methodology for the synthesis of 4-ethynyl-5-iodo-1,2,3-triazoles from iodobuta-1,3-diynes was shown by us [35,40]. The choice of methyl 2-azidoacetate **7** as the dipolarophile caused by the potential ability of derivatization of the ester group. It has been shown that cycloaddition proceeds strictly regioselectively, giving only 5-iodo-1,2,3-iodotriazoles (Scheme 2). Adducts **8a**–**c** were obtained in good yields. 

**Scheme 3:**
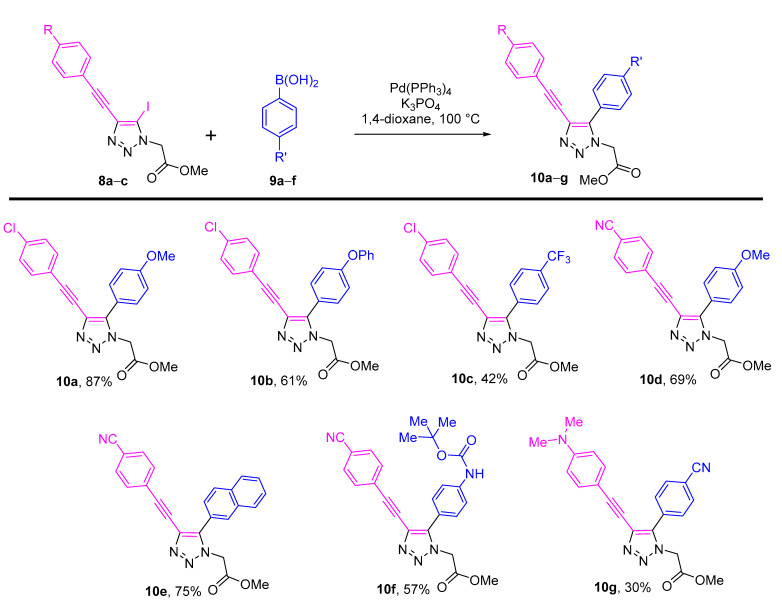
Suzuki-Miyaura cross-coupling of 5-iodo-1,2,3-triazoles **8a**–**c** with boronic acids **9a**–**f**.

Next, we involved the adducts obtained in the Suzuki-Miyaura cross-coupling reaction with boronic acids **9a**–**f** using Pd(PPh_3_)_4_/K_3_PO_4_ catalytic system. The reaction occurs in 1,4-dioxane at 100 °C until the complete conversion of starting 4-ethynyl-5-iodo-1,2,3-triazoles **8a**–**c**. The 5-aryl-4-arylethynyl-1*H*-1,2,3-triazoles **10a**–**g** were prepared in all cases with yields from moderate to high (Scheme 3). It was noticeable that yields of triazoles **10** were significantly higher for the reactions with boronic acids containing electron donating substituents (up to 87% for triazole **10a**). At the same time, in the reactions with boronic acids **9c** and **9f**, containing strong electron-withdrawing groups: trifluoromethyl- and cyano-group, respectively, we were able to obtain 5-aryl-4-ethynyl-1*H*-1,2,3-triazoles **10c,g** with only moderate yield due to complex unidentifiable mixtures of by-products were obtained in addition to the target products. Moreover, using CN-substituted boronic acid **10g** led to a significant increasing in the reaction time (up to 17 h, in other cases full conversion of **8** was achieved from 4 to 7 h). Thus, we synthesized a range of conjugated donor-acceptor systems, promising further investigation of their photophysical properties.

**Figure 1:**
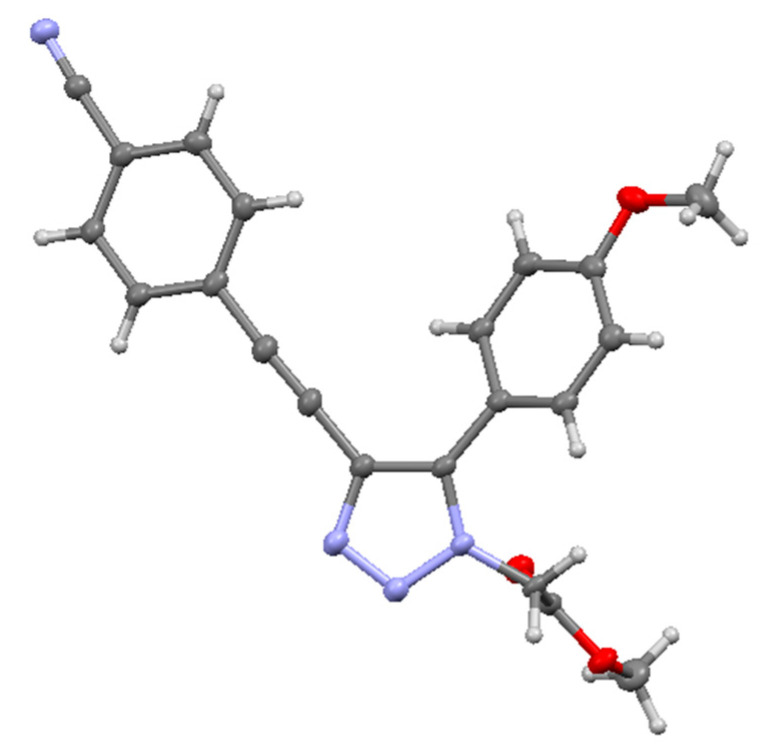
Single-crystal X-ray structure of compound **10d**.

X-ray diffraction analysis data obtained for compound **10d** is shown in Figure 1. It should be noticed that the aryl ring or arylethynyl system lie almost in the same plane as the triazole ring; however, the aryl ring at 5-th position is turned out of the plane. The dihedral angle between the 4-methoxyphenyl and 4 triazole rings was found to be 17.3°. 

### 2.2. Investigation of Optical Properties 

**Figure 2:**
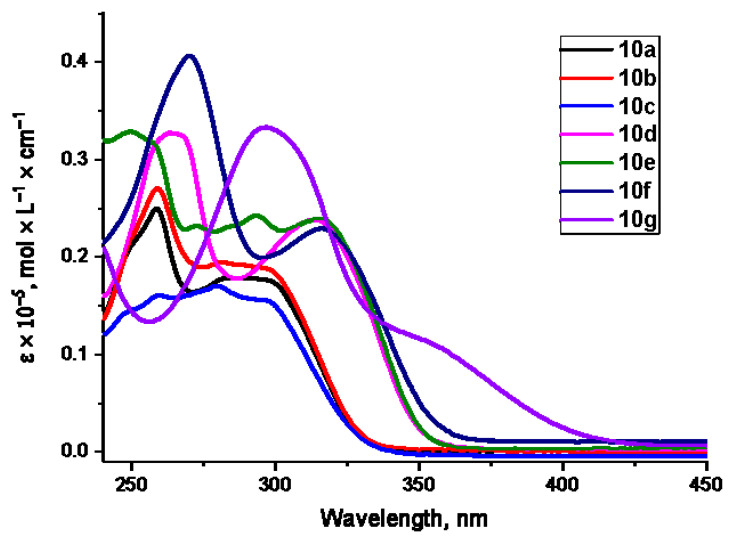
Absorption spectra of **10a**–**g** in THF, C = 1 × 10^–5^ M.

First, the UV-vis absorption spectra were obtained for all target molecules **10a**–**g** (1 × 10^−5^ M solutions in THF) (Figure 2). The obtained spectra differ significantly in the nature of transitions; however, all of them have maximums of absorption in the region from 240 to 450 nm. A significant hyperchromic effect is observed for **10d**–**g**, containing cyano- or dimethylamino substituent in arylethynyl fragment compared with compounds **10a**–**c**. Moreover, a significant bathochromic shift can be observed for **10g**, the only synthesized compound, carrying electron-donating dimethylamino substituent in arylethynyl fragment.

**Figure 3:**
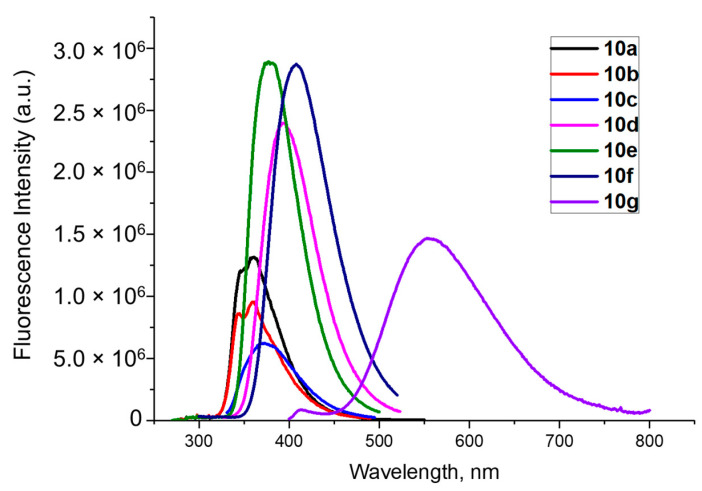
Emission spectra of **10a**–**g** in THF, C = 1 × 10^−6^ M.

Next, the fluorescence spectra of **10a**–**g** were obtained for 1 × 10^−6^ M solutions in THF at room temperature (Figure 3). It was shown that all synthesized compounds exhibited an ability to luminesce and had emission maxima from 361 to 553 nm. Wherein, the wavelength of fluorescence dramatically depended on the nature of the substituent at *C^4^*-position of triazole ring. For **10g**, containing electron-donating dimethylamino group, the significant bathochromic shift was observed. On the other hand, a hypsochromic shift can be noted for compounds, containing chlorine: **10a** had emission a maximum at 361 nm, while **10d**, containing cyano group instead of chlorine had maximum at 394 nm. In addition, the presence of cyano group in arylethynyl fragment led to the hyperchromic effect for compounds **10d**–**f**. 

To quantify the observed fluorescence, we measured absolute quantum yield (*Φ_F_*) for **10a**–**g** in THF solutions. Summarized data on optical properties for compounds **10a**–**g** in THF solutions are given in Table 1. The lowest values of quantum yields were obtained for compounds **10a**–**c**, containing chlorine in arylethynyl fragment. The highest values were obtained for **10e** and **10f**, containing (4-cyanophenyl)ethynyl moiety in *C*^4^-position of triazole ring. However, for [4-(dimethylamino)phenyl]ethynyl substituted triazole **10g**, the *Φ_F_* lower in comparison with **10e** and **10f**, it was also high enough to make this structure interesting for further investigation. The lifetimes of the excited state were also measured for all synthesized compounds. The obtained values of the lifetimes were characteristic for fluorescence.

Another important indicator of the potential use of the compound as fluorescent dyes is the Stokes shift. Most of the synthesized compounds, except **10c**, had the values of the Stokes shift more than 10^4^ cm^−1^. The highest value (15815 cm^−1^) was obtained for compound **10g**. 

**Figure 4:**
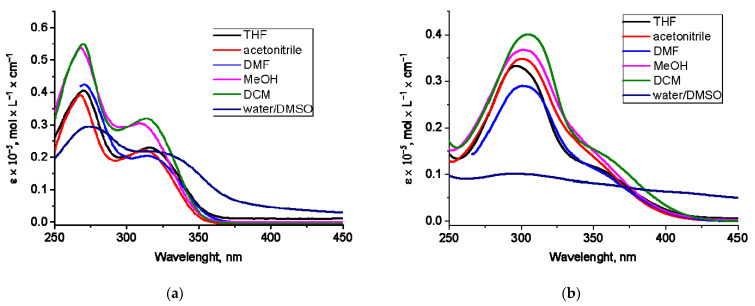
Absorption spectrum of **10f** (**a**) and **10g** (**b**) in various solvents, C = 1 × 10^−5^ M.

**Figure 5:**
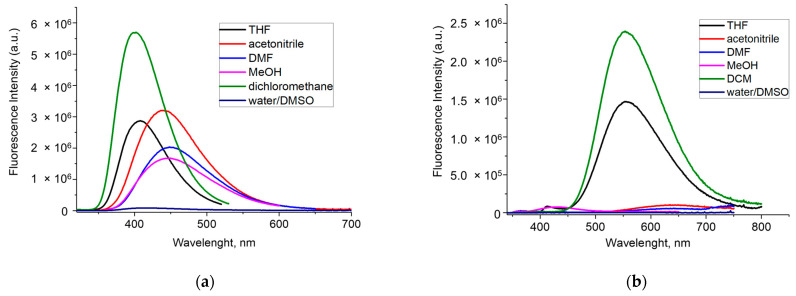
Emission spectra of **10f** (**a**) and **10g** (**b**) in various solvents, C = 1 × 10^−6^ M.

Thus, according to the data obtained on quantum yields and Stokes shifts, the most promising perspective for further investigations are compounds **10f** and **10g**. So, we investigated solvatochromic effects for these two compounds. The absorption and emission spectra were obtained additionally in five solvents: acetonitrile, DMF, methanol, dichloromethane, and in mixture water/DMSO (concentration of DMSO ≤ 1%) (Figure 4 and Figure 5, correspondingly). For the absorption spectra (Figure 4), the nature of the solvent did not have much impact on the values of the main maximums. The intensity of the absorption was the highest in DCM and methanol for both considered compounds and the lowest for the mixture water/DMSO 99:1.

At the same time, the solvents’ nature had a significantly greater effect on the shape of the fluorescence spectra (Figure 5). Wherein, this effect was not the same for **10f** and **10g**. However, the maximal intensity of the fluorescence was observed for the dichloromethane solution, which had the lowest polarity from the considered solvents. In this case, the values of the emission maximum were close to them in THF. The significant hypochromic effect was observed for the mixture water/DMSO 99.9:0.1. 

Summarized data on optical properties for compounds **10f** and **10g** in considered solvents provided in Table 2. For compound **10f** the use of polar protic (MeOH) or aprotic (acetonitrile, DMF) solvents led to bathochromic shift and increasing of the Stokes shifts. Wherein, the intensity of fluorescence is comparable to weakly polar solvents. For **10g,** using polar aprotic solvents also led to bathochromic shift and increasing of the Stokes shifts; however, the intensity of fluorescence was significantly decreased. The use of methanol leads to hypsochromic shift and to decrease fluorescence intensity. The quantum yield of **10f** rises with increasing in the next row of the solvents DMF > dichloromethane > acetonitrile > MeOH > water/DMSO. The lowest fluorescence quantum yield of **10g** was observed in the in a polar, protic solvent, MeOH. The highest fluorescence quantum yield was observed in a polar, aprotic solvent, DCM (Table 2).

### 2.3. Biological Study of 5-aryl-4-arylethynyl-1H-1,2,3-triazoles

As it was discussed in the introduction, a wide range of triazole derivatives possesses valuable pharmacological properties. So, we investigated the antimicrobial activity of compounds **10a**–**g** against Gram-positive bacteria *Staphylococcus aureus*, Gram-negative *Escherichia coli,* and yeast *Candida albicans*. All compounds **10** turned out to be inactive in relation to investigated bacterial culture. In the study of the antifungal effect against the yeast *Candida albicans*, it was found that the compounds have a weak antifungal effect.

**Figure 6:**
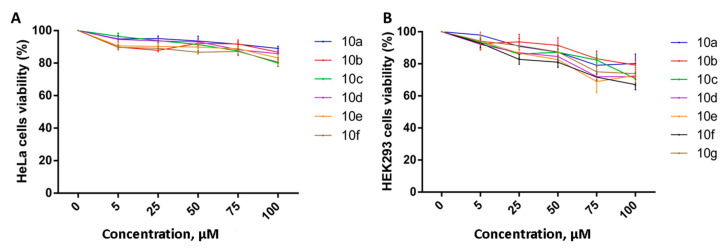
Dose-response curves for HeLa (**A**) and HEK293 (**B**) cells incubated for 24 with different concentrations of the examined compounds. Data points represent mean ± SD for 4 biological replicates.

The MTT test allowed assessing of the cytotoxicity 5-aryl-4-arylethynyltriazoles **10a**–**f** on two distinct cell lines HEK293 and HeLa. The examined compounds did not show any significant cytotoxic effect on both cell lines at concentrations lower than 50 uM (Figure 6).

Thereby, primary screening of biological properties shows the low toxification of the obtained 5-aryl-4-arylethynyl-1*H*-1,2,3-triazoles that makes them promising candidates for the further development of fluorescent labels for cytological studies.

## 3. Materials and Methods

### 3.1. General Information

Solvents and reagents used for reactions were purchased from commercial suppliers. Catalyst Pd(PPh_3_)_4_ was purchased from Sigma-Aldrich (München, Germany). Solvents were dried under standard conditions; chemicals were used without further purification. CuI(PPh_3_)_3_ [41] and 1-iodobuta-1,3-diynes **6a**–**c [35]** were synthesized using known procedures. Evaporation of solvents and concentration of reaction mixtures were performed in vacuum at 35 °C on a rotary evaporator. Thin-layer chromatography (TLC) was carried out on silica gel plates (Silica gel 60, F254, Merck, Darmstadt, Germany) with detection by UV. Melting points (mp) determined were uncorrected. ^1^H and ^13^C NMR spectra (Supplementary Materials) were recorded at 400 and 100 MHz or 126 MHz, respectively, at 25 °C in CDCl_3_ without the internal standard using a 400 MHz Avance spectrometer and 500 MHz Bruker Avance III (Bruker, Billerica, MA, USA). The ^1^H-NMR data were reported as chemical shifts (δ), multiplicity (s, singlet; d, doublet; t, triplet; q, quartet; m, multiplet), coupling constants (*J*, given in Hz), and number of protons. The ^13^C NMR data were reported as the chemical shifts (δ) with coupling constant *J*(C–F) for F-containing compounds. Chemical shifts for ^1^H and ^13^C were reported as values (ppm) and referenced to residual solvent (δ = 7.26 ppm for ^1^H; δ = 77.16 ppm for ^13^C–for spectra in CDCl_3_). High resolution mass spectra (HRMS) were determined using electrospray ionization (ESI) in the mode of positive ion registration with a Bruker microTOF mass analyzer (Billerica, MA, USA). UV–vis spectra for solutions of all compounds were recorded on a UV-1800 spectrophotometer (Shimadzu, Kyoto, Japan) at room temperature. Fluorescence spectra for the same solutions were recorded on a FluoroMax-4 spectrofluorometer (Horiba Scientific, Glasgow, Scotland) at room temperature. Data for **10d** were collected using an XtaLAB SuperNova diffractometer (Rigaku Oxford Diffraction, Tokyo, Japan) equipped with an HyPix3000 CCD area detector operated with monochromated microfocused CuKα radiation (λ[CuKα] = 1.54184 Å). All the data were integrated and corrected for background, Lorentz, and polarization effects by means of the CrysAlisPro (Tokyo, Japan) [42] program complex. Absorption correction was applied using the empirical spherical model within the CrysAlisPro program complex using spherical harmonics, implemented in the SCALE3 ABSPACK scaling algorithm. The unit-cell parameters were refined by the least-squares techniques. The structures were solved by direct methods and refined using the SHELX [43] program incorporated in the OLEX2 [44] program package.

### 3.2. Synthetic Methods and Analytic Data of Compounds

#### 3.2.1. Synthesis of Methyl 2-azidoacetate **7**

Azide **7** was obtained according to [45]. The methyl 2-chloroacetate (2.17 g, 20 mmol) was mixed with NaN_3_ (2.6 g, 40 mmol) and TBAHS (0.679 g, 2 mmol) in a mixture of water (10 mL) and DCM (10 mL). The reaction was allowed to stir at room temperature for 25 h. Then, the aqueous layer was removed and the organic layer was washed with water (3 × 10 mL), dried over Na_2_SO_4_, and filtered. The solvent was removed under reduced pressure to give 1.4 g (61%) of azide **7**. The characterization data for this compound matched that of a previous report [46].

#### 3.2.2. General Procure for the CuAAC.

An azide (1.00 equiv), CuI(PPh_3_)_3_ (5 mol%), and 2,6-lutidine (4 mol%) were consistently added in a screw vial to 1-iodobuta-1,3-diyne (1.00 equiv). The thick resulting mixture was vigorously stirred for 5−24 h at room temperature. After completion of the reaction (TLC control), the reaction mixture was diluted with CH_2_Cl_2_ and a saturated aqueous solution of NH_4_Cl. The reaction mixture was shaken; the organic layer was separated, dried over anhydrous Na_2_SO_4_, and concentrated under reduced pressure to yield the crude product, which was purified by column chromatography on silica gel.

*Methyl 2-{4-[(4-chlorophenyl)ethynyl]-5-iodo-1H-1,2,3-triazol-1-yl}acetate* (**8a**). This compound was synthesized according to the general procedure for the CuAAC from 1-iodobuta-1,3-diyne **6a** (346 mg, 1.21 mmol) and azide **7** (139 mg, 1.21 mmol). Reaction time—20 h. The crude product was purified by column chromatography (eluent: hexane/EtOAc = 5:1 → 2:1) to afford a white solid (344 mg, 71% yield): mp 139−140 °C; ^1^H NMR (400 MHz, CDCl_3_) δ 7.55–7.48 (m, 2H_Ar_), 7.38–7.31 (m, 2H_Ar_), 5.21 (s, 2H, CH_2_), 3.82 (s, 3H, CH_3_). ^13^C NMR (101 MHz, CDCl_3_) δ 165.7, 138.6, 135.4, 133.1, 129.0, 120.6, 94.1, 85.8, 79.2, 53.4, 51.7. HRMS ESI [M + Na]^+^ calcd for C_13_H_9_ClIN_3_O_2_Na^+^ 423.9320, found 423.9316.

*Methyl 2-{4-[(4-cyanophenyl)ethynyl]-5-iodo-1H-1,2,3-triazol-1-yl}acetate* (**8b**). This compound was synthesized according to the general procedure for the CuAAC from 1-iodobuta-1,3-diyne **6b** (50 mg, 0.18 mmol) and azide **7** (21 mg, 0.18 mmol). Reaction time—24 h. The crude product was purified by column chromatography (eluent: hexane/acetone = 3:1) to afford a white solid (62 mg, 87% yield): mp 203−205 °C (decomposition); ^1^H NMR (400 MHz, CDCl_3_) δ 7.70–7.65 (m, 4H_Ar_), 5.23 (s, 2H, CH_2_), 3.84 (s, 3H, CH_3_). ^13^C NMR (126 MHz, CDCl_3_) δ 165.6, 138.1, 132.4, 132.3, 127.0, 118.4, 112.6, 93.4, 86.4, 82.4, 53.5, 51.7. HRMS ESI [M + Na]^+^ calcd for C_14_H_9_IN_4_O_2_Na^+^ 414.9662, found 414.9660.

*Methyl 2-{4-[(4-(dimethylamino)phenyl)ethynyl]-5-iodo-1H-1,2,3-triazol-1-yl}acetate* (**8c**). This compound was synthesized according to the general procedure for the CuAAC from 1-iodobuta-1,3-diyne **6c** (86 mg, 0.29 mmol) and azide **7** (33 mg, 0.29 mmol). Reaction time—5 h. The crude product was purified by column chromatography (eluent: hexane/EtOAc = 3:1 → 1:1) to afford a white solid (82 mg, 69% yield): mp 202−203 °C (decomposition); ^1^H NMR (400 MHz, CDCl_3_) δ 7.46 (d, *J* = 8.8 Hz, 2H_Ar_), 6.67 (d, *J* = 8.8 Hz, 2H_Ar_), 5.20 (s, 2H, CH_2_), 3.82 (s, 3H, CH_3_), 3.00 (s, 6H, CH_3_). ^13^C NMR (126 MHz, CDCl_3_) δ 165.8, 150.9, 139.7, 133.2, 111.9, 108.9, 96.8, 84.6, 76.3, 53.3, 51.7, 40.3. HRMS ESI [M + Na]^+^ calcd for C_15_H_15_IN_4_O_2_Na^+^ 433.0132, found 433.0128.

#### 3.2.3. General Procure for the Suzuki-Miyaura cross-coupling.

5-Iodo-1*H*-1,2,3-triazoles **8a−c** (1 equiv), ArB(OH)_2_ **9a−f** (2 equiv), K_3_PO_4_ (2 equiv), and Pd(PPh_3_)_4_ (5 mol%) were placed in a vial. The vial was sealed, and the mixture was evacuated and flushed with Ar several times. 1,4-Dioxane (0.08 M) was added, and the vial with the reaction mixture was placed in a preheated IKA Dry Block Heater (100 °C) and stirred for 4−17 h (TLC control). After cooling to rt, the reaction mixture was filtered through a pad of silica gel and washed with CH_2_Cl_2_. Solvents were removed under reduced pressure, and the crude product was purified by column chromatography on silica gel.

*Methyl 2-{4-[(4-chlorophenyl)ethynyl]-5-(4-methoxyphenyl)-1H-1,2,3-triazol-1-yl}acetate* (**10a**). This compound was synthesized according to the general procedure from 5-iodo-1*H*-1,2,3-triazole **8a** (150 mg, 0.374 mmol) and boronic acid **9a** (114 mg, 0.748 mmol). Reaction time—4 h. The crude product was purified by column chromatography (eluent: hexane/EtOAc = 2:1) to afford a white solid (125 mg, 87% yield): mp 104−105 °C (benzene); ^1^H NMR (400 MHz, CDCl_3_) δ 7.47–7.42 (m, 2H_Ar_), 7.41–7.36 (m, 2H_Ar_), 7.31–7.27 (m, 2H_Ar_), 7.11–6.99 (m, 2H_Ar_), 5.11 (s, 2H, CH_2_), 3.88 (s, 3H, CH_3_), 3.78 (s, 3H, CH_3_). ^13^C NMR (101 MHz, CDCl_3_) δ 166.9, 161.2, 140.5, 134.9, 133.0, 130.5, 129.5, 128.8, 121.1, 117.5, 114.9, 92.0, 80.3, 55.6, 53.2, 49.7. HRMS ESI [M + Na]^+^ calcd for C_20_H_16_ClN_3_O_3_Na^+^ 404.0772, found 404.0771. 

*Methyl 2-{4-[(4-chlorophenyl)ethynyl]-5-(4-phenoxyphenyl)-1H-1,2,3-triazol-1-yl}acetate* (**10b**). This compound was synthesized according to the general procedure from 5-iodo-1*H*-1,2,3-triazole **8a** (150 mg, 0.374 mmol) and boronic acid **9b** (160 mg, 0.748 mmol). Reaction time—4 h. The crude product was purified by column chromatography (eluent: hexane/EtOAc = 2:1) to afford a colorless oil (102 mg, 61% yield); ^1^H NMR (400 MHz, CDCl_3_) δ 7.50–7.45 (m, 2H_Ar_), 7.44–7.37 (m, 4H_Ar_), 7.32–7.27 (m, 2H_Ar_), 7.24–7.18 (m, 1H_Ar_), 7.14–7.07 (m, 4H_Ar_), 5.12 (s, 2H, CH_2_), 3.79 (s, 3H, CH_3_). ^13^C NMR (101 MHz, DMSO) δ 166.9, 159.7, 155.8, 140.1, 135.0, 133.0, 130.7, 130.2, 129.6, 128.9, 124.7, 121.0, 120.2, 119.5, 118.5, 92.2, 80.2, 53.3, 49.7. HRMS ESI [M + Na]^+^ calcd for C_25_H_18_ClN_3_O_3_Na^+^ 466.0929, found 466.0926.

*Methyl 2-{4-[(4-chlorophenyl)ethynyl]-5-[4-(trifluoromethyl)phenyl]-1H-1,2,3-triazol-1-yl}acetate* (**10c**). This compound was synthesized according to the general procedure from 5-iodo-1*H*-1,2,3-triazole **8a** (80 mg, 0.199 mmol) and boronic acid **9c** (75 mg, 0.398 mmol). Reaction time—7 h. The crude product was purified by column chromatography (eluent: hexane/acetone = 5:1) to afford a yellow oil (35 mg, 42% yield); ^1^H NMR (400 MHz, CDCl_3_) δ 7.82 (d, *J* = 8.2 Hz, 2H_Ar_), 7.68 (d, *J* = 8.2 Hz, 2H_Ar_), 7.42–7.35 (m, 2H_Ar_), 7.34–7.28 (m, 2H_Ar_), 5.14 (s, 2H, CH_2_), 3.79 (s, 3H, CH_3_). ^13^C NMR (101 MHz, CDCl_3_) δ 166.6 (C), 140.0 (C), 135.3 (C), 133.0 (2CH), 132.45 (q, ^2^*J*_C-F_ = 33.0 Hz, C), 130.3 (C), 129.6 (2CH), 129.4 (C), 129.0 (2CH), 126.43 (q, ^3^*J*_C-F_ = 3.7 Hz, 2CH), 123.73 (q, ^1^*J*_C-F_ = 272.6 Hz, C), 120.6 (C), 92.7 (C), 79.4 (C), 53.4 (CH_3_), 49.8 (CH_2_). HRMS ESI [M + Na]^+^ calcd for C_20_H_13_ClF_3_N_3_O_2_Na^+^ 442.0541, found 442.0539. Appropriate crystals for X-Ray analysis were obtained from acetonitrile solution. Crystallographic data for **10c** were deposited with the Cambridge Crystallographic Data Centre, no. CCDC 2077046.

*Methyl 2-{4-[(4-cyanophenyl)ethynyl]-5-(4-methoxyphenyl)-1H-1,2,3-triazol-1-yl}acetate* (**10d**). This compound was synthesized according to the general procedure from 5-iodo-1*H*-1,2,3-triazole **8b** (90 mg, 0.230 mmol) and boronic acid **9a** (70 mg, 0.460 mmol). Reaction time—7 h. The crude product was purified by column chromatography (eluent: hexane/acetone = 5:1) to afford a colorless crystals (59 mg, 69% yield): mp 146−147 °C (acetonitrile); ^1^H NMR (400 MHz, CDCl_3_) δ 7.63–7.58 (m, 2H_Ar_), 7.56–7.51 (m, 2H_Ar_), 7.46–7.41 (m, 2H_Ar_), 7.09–7.03 (m, 2H, CH_Ar_), 5.12 (s, 2H, CH_2_), 3.89 (s, 3H, CH_3_), 3.79 (s, 3H, CH_3_). ^13^C NMR (101 MHz, CDCl_3_) δ 166.8, 161.4, 141.1, 132.2, 132.2, 130.5, 128.9, 127.5, 118.5, 117.3, 115.0, 112.1, 91.5, 83.8, 55.6, 53.3, 49.7. HRMS ESI [M + Na]^+^ calcd for C_21_H_16_N_4_O_3_Na^+^ 395.1115, found 395.1110. 

*Methyl 2-{4-[(4-cyanophenyl)ethynyl]-5-(naphthalen-2-yl)-1H-1,2,3-triazol-1-yl}acetate* (**10e**). This compound was synthesized according to the general procedure from 5-iodo-1*H*-1,2,3-triazole **8b** (75 mg, 0.191 mmol) and boronic acid **9d** (66 mg, 0.383 mmol). Reaction time—5 h. The crude product was purified by column chromatography (eluent: hexane/EtOAc = 2:1) to afford a white solid (43 mg, 75% yield): mp 129−131 °C; ^1^H NMR (400 MHz, CDCl_3_) δ 8.05–8.00 (m, 2H_Ar_), 7.96–7.90 (m, 2H_Ar_), 7.66–7.55 (m, 5H_Ar_), 7.53–7.47 (m, 2H_Ar_), 5.20 (s, 2H, CH_2_), 3.78 (s, 3H, CH_3_). ^13^C NMR (101 MHz, CDCl_3_) δ 166.8, 141.3, 133.9, 133.2, 132.2, 132.2, 129.5, 129.5, 129.3, 128.5, 128.2, 128.1, 127.5, 127.4, 125.5, 122.6, 118.5, 112.1, 91.7, 83.6, 53.3, 49.8. HRMS ESI [M + Na]^+^ calcd for C_24_H_16_N_4_O_2_Na^+^ 415.1165, found 415.1163.

*Methyl 2-(5-{4-[(tert-butoxycarbonyl)amino]phenyl}-4-[(4-cyanophenyl)ethynyl]-1H-1,2,3-triazol-1-yl)acetate* (**10f**). This compound was synthesized according to the general procedure from 5-iodo-1*H*-1,2,3-triazole **8b** (80 mg, 0.204 mmol) and boronic acid **9e** (97 mg, 0.408 mmol). Reaction time—5 h. The crude product was purified by column chromatography (eluent: hexane/EtOAc = 2:1) to afford a white solid (53 mg, 57% yield): mp 87−88 °C; ^1^H NMR (400 MHz, CDCl_3_) δ 7.63–7.50 (m, 6H_Ar_), 7.44 (d, *J* = 8.6 Hz, 2H), 6.68 (s, 1H, NH), 5.12 (s, 2H, CH_2_), 3.78 (s, 3H, CH_3_), 1.54 (s, 9H, CH_3_). ^13^C NMR (101 MHz, CDCl_3_) δ 166.8, 152.5, 140.9, 140.7, 132.2, 132.2, 129. 9, 129.0, 127.5, 119.2, 118.9, 118.5, 112.1, 91.5, 83.7, 81. 6, 53.3, 49.7, 28.4. HRMS ESI [M + Na]^+^ calcd for C_25_H_23_N_5_O_4_Na^+^ 480.1642, found 480.1642.

*Methyl 2-[5-(4-cyanophenyl)-4-{[4-(dimethylamino)phenyl]ethynyl}-1H-1,2,3-triazol-1-yl]acetate* (**10g**). This compound was synthesized according to the general procedure from 5-iodo-1*H*-1,2,3-triazole **8b** (96 mg, 0.234 mmol) and boronic acid **9e** (69 mg, 0.468 mmol). Reaction time—17 h. The crude product was purified by column chromatography (eluent: hexane/EtOAc = 2:1 → 1:1) to afford a yellow solid (27 mg, 30% yield): mp 165−166 °C; ^1^H NMR (400 MHz, CDCl_3_) δ 7.86–7.79 (m, 2H_Ar_), 7.74–7.67 (m, 2H_Ar_), 7.37–7.29 (m, 2H_Ar_), 6.66 (d, *J* = 8.7 Hz, 2H_Ar_), 5.14 (s, 2H, CH_2_), 3.78 (s, 3H, CH_3_), 2.99 (s, 6H, CH_3_). ^13^C NMR (101 MHz, CDCl_3_) δ 166.6, 150.7, 137.4, 133.1, 133.0, 131.7, 130.8, 129.7, 118.1, 113.9, 111.9, 108.5, 95.8, 76.2, 53.4, 50.0, 40.3. HRMS ESI [M + Na]^+^ calcd for C_22_H_19_N_5_O_2_Na^+^ 408.1431, found 408.1428.

### 3.3. The Absolute Fluorescence Quantum Yield Measurements

The absolute fluorescence quantum yield was measured on a Horiba Fluorolog-3 spectrometer (Edison, NJ, USA) equipped using an integrating sphere. A xenon lamp coupled to a double monochromator was used as excitation light source. The sample (1 cm quartz cuvette cell with solution in THF) or blank (pure THF) was directly illuminated in the center of the integrating sphere. The optical density of all investigated sample solutions in corresponding solvent did not exceed 0.1 at the luminescence excitation wavelength. Under the same conditions (e.g., excitation wavelength, spectral resolution, temperature), the luminescence spectrum of the sample *Ec*, the luminescence spectrum of the blank *Ea*, the Rayleigh scattering spectrum of the sample *Lc*, and the Rayleigh scattering spectrum of the solvent *La* were measured. The absolute fluorescence quantum yield was determined according to the formula:

*Φ_F_* = *(Ec − Ea)*/*(La − Lc)* (1)

### 3.4. Determination of Minimum Inhibitory Concentration (MIC) 

The antimicrobial activity of 1,2,3-triazoles **10a**–**g** was studied by the method of double serial dilutions. Gram-positive bacteria Staphylococcus aureus ATCC 6538, Gram-negative bacteria Escherichia coli ATCC 25,922, and yeast Candida albicans RCPGU401 were used as test cultures. Initial solutions of 1,2,3-triazoles at a concentration of 1 mg/ml were prepared in 50% aqueous dimethyl sulfoxide due to their limited solubility in water. To obtain a series of dilutions of 1,2,3-triazoles, 1 ml of meat-peptone broth (for bacteria) or Sabouraud Dextrose Broth (for fungi) were added to sterile test tubes. A total of 1 ml of the initial solution of the compound at a concentration of 1 mg/ml was added in the first test tube and a series of consecutive double dilutions was performed. Then, 0.1 ml of microbial inoculate was added to each test tube. The microbial load was 105 CFU/ml for bacteria and 104 CFU/ml for fungi. A liquid culture medium with a suspension of microorganisms without the addition of triazole was the control medium. The samples were incubated for 24 h at 37 °C for bacteria and 48 h at 24 °C for fungi. The presence of growth of test cultures in a liquid medium was determined by the turbidity of the medium. The minimum inhibitory concentrations of the compounds were determined. The experiment was performed under aseptic conditions.

### 3.5. Cell Culture Cultivation and Cytotoxicity Studies

To assess the cytotoxicity, two distinct cell lines were investigated, namely HEK293 and HeLa, due to their different properties and origins. The proportion of viable cells after the exposure to the compounds was determined using the MTT assay [47] by assessing their metabolic activity in the cell culture. HEK293 and HeLa cell cultures were grown in DMEM standard medium supplemented with 10% fetal bovine serum (FBS) at 37 °C in an atmosphere containing 5% CO_2_. The cells were transferred to a 96-well plate (5000 cells per well in 100 μl DMEM + 10% FBS). The plates were incubated for 24 h, and culture medium was replaced with 100 μL DMEM + 10% FBS containing various concentrations of the examined compounds (5, 25, 50, 75, and 100 μM). After 24 h of incubation, 20 μL of MTT solution (3-(4,5-dimethylthiazol-2-yl)-2,5-diphenyltetrazolium bromide at a concentration of 5 mg/ml) was added to the wells. After 3 h of incubation, the medium was removed and 100 μL of DMSO was added to each well. Using BioRad xMark microplate spectrophotometer, the absorbance of the resulting solutions was measured at 570 nm. The obtained values are directly proportional to the number of surviving cells after cultivation in the presence of the examined compounds. The percentage of cell viability in the presence of the examined compounds relative to non-treated cells was calculated.

## 4. Conclusions

The efficiency of the approach combining CuAAC of methyl 2-azidoacetate to iodobuta-1,3-diynes and subsequent Suzuki-Miyaura cross-coupling reaction were demonstrated for synthesis of 5-aryl-4-arylethynyl-1*H*-1,2,3-triazoles. The CuAAC proceeds strictly regioselectively giving 4-arylethynyl-5-iodo-1,2,3-triazoles with 69–87% yield. Subsequent modification of cycloadducts were able to obtain a range of 5-aryl-4-arylethynyl-1*H*-1,2,3-triazoles with the 30–87% yield. The promising photophysical properties were able to demonstrate for all target compounds. The effects of the solvent on fluorescent parameters were demonstrated for two of the most perspective compounds. All 5-aryl-4-arylethynyl-1*H*-1,2,3-triazoles obtained did not show any significant cytotoxic effect on cell lines HEK293 and HeLa and could be considered as candidates for the development of fluorescent labels for bioimaging after additional structural design aimed to maintain high fluorescence intensity in aqueous media. 

## Figures and Tables

**Table 1:** Photophysical parameters of **10a**–**g** (THF solutions).

Compound	λ_abs_, nm	ε	λ_ex_, nm	λ_em_, nm	*Φ_F_*, %	T, ns	Stokes shift, cm^−1^
**10a**	258.5	24,940	258	361	26.2	0.4, 1.5	11,059
**10b**	259	27,055	258	361	18.8	0.2, 0.9	11,059
**10c**	279.5	17,024	279	371	15.5	0.4, 1.5	8888
**10d**	263	32,742	265	394	34.7	1.5	12,355
**10e**	249	32,890	255	377	62.5	0.9, 3.7	12,690
**10f**	270	40,660	270	408	64.4	2.1	12,527
**10g**	296.5	33,319	295	553	36.2	7.1	15,815

**Table 2:** Photophysical parameters **10f** and **10g** in various solvents.

Compound	Solvent	λ_ex_, nm	λ_em_, nm	*Φ_F_*, %	T, ns	Stokes Shift, cm^−1^
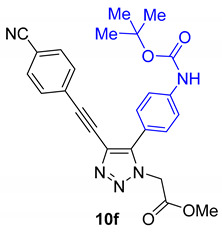	acetonitrile	266	439	27.0	3.5	14,814
	DMF	275	448	38.9	4.2	14,043
	MeOH	268	447	19.4	3.1	14,942
	dichloromethane	269	402	32.3	1.6	12,299
	water/DMSO	273	413	7.8	1.1, 4.5	12,417
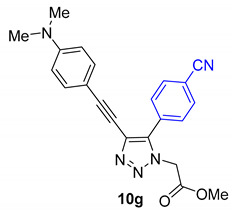	acetonitrile	300	644	1.7	1.4	17,805
	DMF	300	642	0.9	1.7, 6.6	17,757
	MeOH	302	445	-0.05	4.2	10,641
	dichloromethane	306	554	29.0	8.2	14,629
	water/DMSO	286	484	5.3	4.1, 16.2	14,304

## Data Availability

The presented data are available in this article.

## Supplementary Materials

The following are available online, copies of ^1^H, ^13^C, and DEPT NMR spectra for all new compounds; cif file with X-ray data for compound **10d**.

## Sample Availability

Samples of the all compounds are available from the authors.

## References

[1] Nwe K., Brechbiel M.W. (2009). Growing Applications of ‘‘Click Chemistry’’ for Bioconjugation in Contemporary Biomedical Research. Cancer Biother. Radiopharm..

[2] Shiri P., Amani A.M. (2021). A brief overview of catalytic applications of dendrimers containing 1,4-disubstituted-1,2,3-triazoles. Monatsh. Chem..

[3] Shad M.S., Santhini P.V., Dehaen W. (2019). 1,2,3-Triazolium macrocycles in supramolecular chemistry. Beilstein J. Org. Chem..

[4] Foyle É.M., White N.G. (2021). Anion Templated Supramolecular Structures Assembled using 1,2,3-Triazole and Triazolium motifs. Chem Asian J..

[5] Li K., Fong D., Meichsner E., Adronov A. (2021). A Survey of Strain-Promoted Azide–Alkyne Cycloaddition in Polymer Chemistry. Chem. Eur. J..

[6] Tornøe C.W., Cristensen C., Meldal M. (2002). Peptidotriazoles on Solid Phase: [1,2,3]-Triazoles by Regiospecific Copper(I)-Catalyzed 1,3-Dipolar Cycloadditions of Terminal Alkynes to Azides. J. Org. Chem..

[7] Rostovtsev V.V., Green L.G., Fokin V.V., Sharpless K.B. (2002). A Stepwise Huisgen Cycloaddition Process: Copper(I)-Catalyzed Regioselective “Ligation” of Azides and Terminal Alkynes. Angew. Chem. Int. Ed..

[8] Dhameja M., Kumar H., Gupta P. (2020). Chiral Fused 1,2,3-Triazoles: A Synthetic Overview. Asian J. Org. Chem..

[9] Nebra N., García-Álvarez J., Campuzano S., Pingarrón J.M. (2020). Recent Progress of Cu-Catalyzed Azide-Alkyne Cycloaddition Reactions (CuAAC) in Sustainable Solvents: Glycerol, Deep Eutectic Solvents, and Aqueous Media. Molecules.

[10] Fantoni N.Z., El-Sagheer A.H., Brown T. (2021). A Hitchhiker’s Guide to Click-Chemistry with Nucleic Acids. Chem. Rev..

[11] Bonandi E., Christodoulou M.S., Fumagalli G., Perdicchia D., Rastelli G., Passarella D. (2017). The 1,2,3-triazole ring as a bioisostere in medicinal chemistry. Drug Discov. Today.

[12] Bozorov K., Zhaoa J., Aisa H.A. (2019). 1,2,3-Triazole-containing hybrids as leads in medicinal chemistry: A recent overview. Bioorg Med. Chem..

[13] Rani A., Singh G., Singh A., Maqbool U., Kaur G., Singh J. (2020). CuAAC-ensembled 1,2,3-triazole-linked isosteres as pharmacophores in drug discovery: Review. RSC Adv..

[14] Kumar S., Sharma B., Mehra V., Kumar V. (2021). Recent accomplishments on the synthetic/biological facets of pharmacologically active 1*H*-1,2,3-triazoles. Eur. J. Med. Chem..

[15] Xu Z. (2020). 1,2,3-Triazole-containing hybrids with potential antibacterial activity against methicillin-resistant Staphylococcus aureus (MRSA). Eur. J. Med. Chem..

[16] Feng L.-S., Zheng M.-J., Zhao F., Liu D. (2021). 1,2,3-Triazole hybrids with anti-HIV-1 activity. Arch Pharm..

[17] Mashayekh K., Shiri P. (2019). An Overview of Recent Advances in the Applications of Click Chemistry in the Synthesis of Bioconjugates with Anticancer Activities. ChemistrySelect.

[18] Xu Z., Zhao S.-J., Liu Y. (2019). 1,2,3-Triazole-containing hybrids as potential anticancer agents: Current developments, action mechanisms and structure-activity relationships. Eur. J. Med. Chem..

[19] Brunel D., Dumur F. (2020). Recent advances in organic dyes and fluorophores comprising a 1,2,3-triazole moiety. New J. Chem..

[20] Duan T., Fan K., Zhong C., Chen X., Peng T., Qin J. (2012). Triphenylamine-based organic dyes containing a 1,2,3-triazole bridge for dye-sensitized solar cells via a ‘Click’ reaction. Dyes Pigm..

[21] Yen Y.-S., Hsu J.-L., Ni J.-S., Lin J.T. (2021). Influence of various dithienoheterocycles as conjugated linker in Naphtho [2,3-*d*] [1,2,3]triazole-based organic dyes for dye-sensitized solar cells. Dyes Pigm..

[22] Jarowski P.D., Wu Y.-L., Schweizer W.B., Diederich F. (2008). 1,2,3-Triazoles as Conjugative π-Linkers in Push-Pull Chromophores: Importance of Substituent Positioning on Intramolecular Charge-Transfer. Org. Lett..

[23] Zhu Y., Guang S., Su X., Xu H., Xu D. (2013). Dependence of the intramolecular charge transfer on molecular structure in triazole bridge-linked optical materials. Dyes Pigm..

[24] Kautny P., Gloöcklhofer F., Kader T., Mewes J.-M., Stöger B., Froöhlich J., Lumpi D., Plasser F. (2017). Charge-transfer states in triazole linked donor–acceptor materials: Strong effects of chemical modification and solvation. Phys. Chem. Chem. Phys..

[25] Kautny P., Bader D., Stöger B., Reider G.A., Fröhlich J., Lumpi D. (2016). Structure–Property Relationships in Click-Derived Donor–Triazole–Acceptor Materials. Chem. Eur. J..

[26] Shi J., Liu L., He J., Meng X., Guo Q. (2007). Facile Derivatization of Pyridyloxazole-type Fluorophore via Click Chemistry. Chem. Lett..

[27] Šišuļins A., Bucevičius J., Tseng Y.-T., Novosjolova I., Traskovskis K., Bizdēna Ē., Chang H.-T., Tumkevičius S., Turks M. (2019). Synthesis and fluorescent properties of N(9)-alkylated 2-amino-6-triazolylpurines and 7-deazapurines. Beilstein J. Org. Chem..

[28] Cornec A.-S., Baudequin C., Fiol-Petit C., Plé N., Dupas G., Ramondenc Y. (2013). One “Click” to Access Push–Triazole–Pull Fluorophores Incorporating a Pyrimidine Moiety: Structure–Photophysical Properties Relationships. Eur. J. Org. Chem..

[29] Tsyrenova B., Nenajdenko V. (2020). Synthesis and Spectral Study of a New Family of 2,5-Diaryltriazoles Having Restricted Rotation of the 5-Aryl Substituent. Molecules.

[30] Ahmed F., Xiong H. (2021). Recent developments in 1,2,3-triazole-based chemosensors. Dye. Pigment..

[31] Bryant J.J., Bunz U.H.F. (2013). Click To Bind: Metal Sensors. Chem. Asian J..

[32] Danilkina N.A., Govdi A.I., Balova I.A. (2020). 5-Iodo-1H-1,2,3-triazoles as Versatile Building Blocks. Synthesis.

[33] Hein J.E., Fokin V.V. (2010). Copper-catalyzed azide–alkyne cycloaddition (CuAAC) and beyond: New reactivity of copper(I) acetylides. Chem. Soc. Rev..

[34] Wei F., Wang W., Ma Y., Tung C.-H., Xu Z. (2016). Regioselective synthesis of multisubstituted 1,2,3-triazoles: Moving beyond the copper-catalyzed azide–alkyne cycloaddition. Chem. Commun..

[35] Govdi A.I., Danilkina N.A., Ponomarev A.V., Balova I.A. (2019). 5-Iodo-1H-1,2,3-triazoles as Versatile Building Blocks. J. Org. Chem..

[36] Favorskii A.E., Skosarevskii M.P. (1900). About the Reaction of Powdered Potassium Hydroxide on a Mixture of Phenylacetylene and Acetone. Zh. Russ. Khim. Ob-va.

[37] Danilkina N.A., Vasileva A.A., Balova I.A. (2020). A.E.Favorskii’s scientific legacy in modern organic chemistry: Prototropic acetylene–Allene isomerization and the acetylene zipper reaction. Russ. Chem. Rev..

[38] Sonogashira K., Tohda Y., Hagihara N. (1975). A Convenient Synthesis of Acetylenes: Catalytic Substitutions of Acetylenic Hydrogen with Bromoalkenes, Iodoarenes and Bromopyridines. Tetrahedron Lett..

[39] Lal S., Rzepa H.S., Díez-González S. (2014). Catalytic and Computational Studies of N-Heterocyclic Carbene or Phosphine-Containing Copper (I) Complexes for the Synthesis of 5-Iodo-1,2,3-Triazoles. ACS Catal..

[40] Danilkina N.A., D’Yachenko A.S., Govdi A.I., Khlebnikov A.F., Kornyakov I.V., Bräse S., Balova I.A. (2020). Intramolecular Nicholas Reactions in the Synthesis of Heteroenediynes Fused to Indole, Triazole, and Isocoumarin. J. Org. Chem..

[41] Maini L., Braga D., Mazzeo P.P., Ventura B. (2012). Polymorph and isomer conversion of complexes based on CuI and PPh_3_ easily observed *via* luminescence. Dalton Trans..

[42] (2015). CrysAlisPro.

[43] Sheldrick G.M. (2015). Crystal structure refinement with SHELXL. Acta Crystallogr. Sect. C Struct. Chem..

[44] Dolomanov O.V., Bourhis L.J., Gildea R.J., Howard J.A.K., Puschmann H. (2009). OLEX2: A complete structure solution, refinement and analysis program. J. Appl. Crystallogr..

[45] Zheng H., McDonald R., Hall D.G. (2010). Boronic Acid Catalysis for Mild and Selective [3+2] Dipolar Cycloadditions to Unsaturated Carboxylic Acids. Chem. Eur. J..

[46] Lüth A., Löwe W. (2008). Syntheses of 4-(indole-3-yl)quinazolines–A new class of epidermal growth factor receptor tyrosine kinase inhibitors. Eur. J. Med. Chem..

[47] Van Meerloo J., Kaspers G.J., Cloos J. (2011). Cell sensitivity assays: The MTT assay. Methods Mol Biol..

