# Supplementary material for: Design and Synthesis of New 5-aryl-4-Arylethynyl-1H-1,2,3-triazoles with Valuable Photophysical and Biological Properties
Source: Molecules. 2021 May 10;26(9):2801. doi: 10.3390/molecules26092801 (PMC8126154; doi:10.3390/molecules26092801)

Supplementary Information

Design and synthesis of new 5-aryl-4-arylethynyl-1*H*-1,2,3-triazoles with valuable photophysical and biological properties

Mariia M. Efremova [1], Anastasia I. Govdi [1,*], Valeria V. Frolova [2], Andrey M. Rumyantsev [3] and Irina A. Balova [1,*]

[1] Institute of Chemistry, Saint Petersburg State University (SPbU), Universitetskaya nab. 7/9, Saint Petersburg 199034, Russia; m.efremova@2012.spbu.ru (M.M.E.); a.govdi@spbu.ru (A.I.G.)

[2] Saint Petersburg State Chemical Pharmaceutical University (SPCPU), 14A Professor Popov Str., Saint Petersburg 197376, Russia; zhilyaeva.valeriya@pharminnotech.com (V.V.F.)

[3] Department of Genetics and Biotechnology Saint Petersburg State University (SPbU), Universitetskaya nab. 7/9, Saint Petersburg 199034, Russia; rumyantsev-am@mail.ru (A.M.R.)

* Correspondence i.balova@spbu.ru; a.govdi@spbu.ru; Tel.: +7-812-428-6733

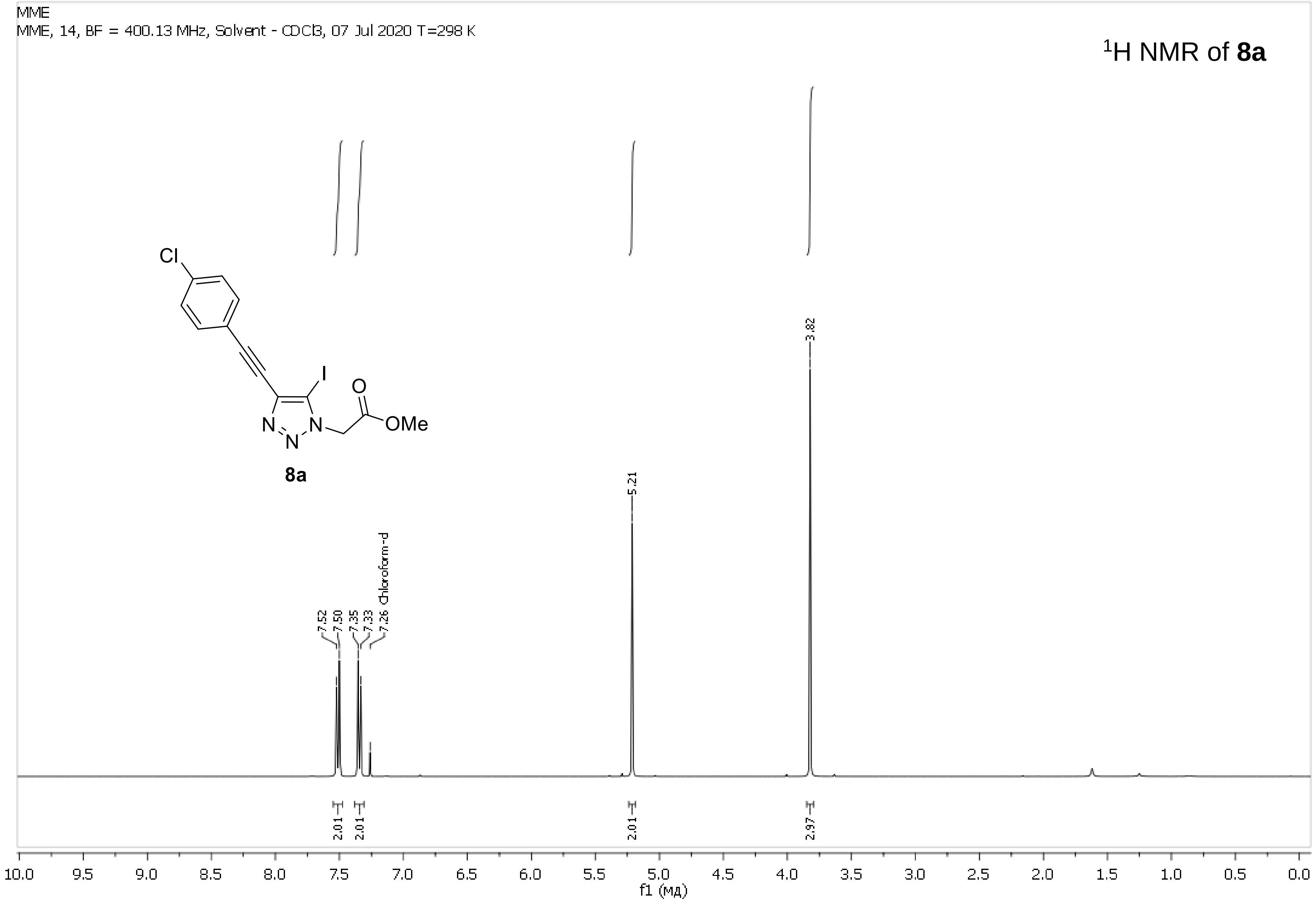
MME
MME, 14, BF = 400.13 MHz, Solvent - CDCl3, 07 Jul 2020 T=298 K
1H NMR of 8a
Cl
I
O
OMe
N
N
N
8a
7.52
7.50
7.35
7.33
7.26 Chloroform-d
5.21
3.82
2.01
2.01
2.01
2.97
10.0 9.5 9.0 8.5 8.0 7.5 7.0 6.5 6.0 5.5 5.0 4.5 4.0 3.5 3.0 2.5 2.0 1.5 1.0 0.5 0.0
f1 (мд)

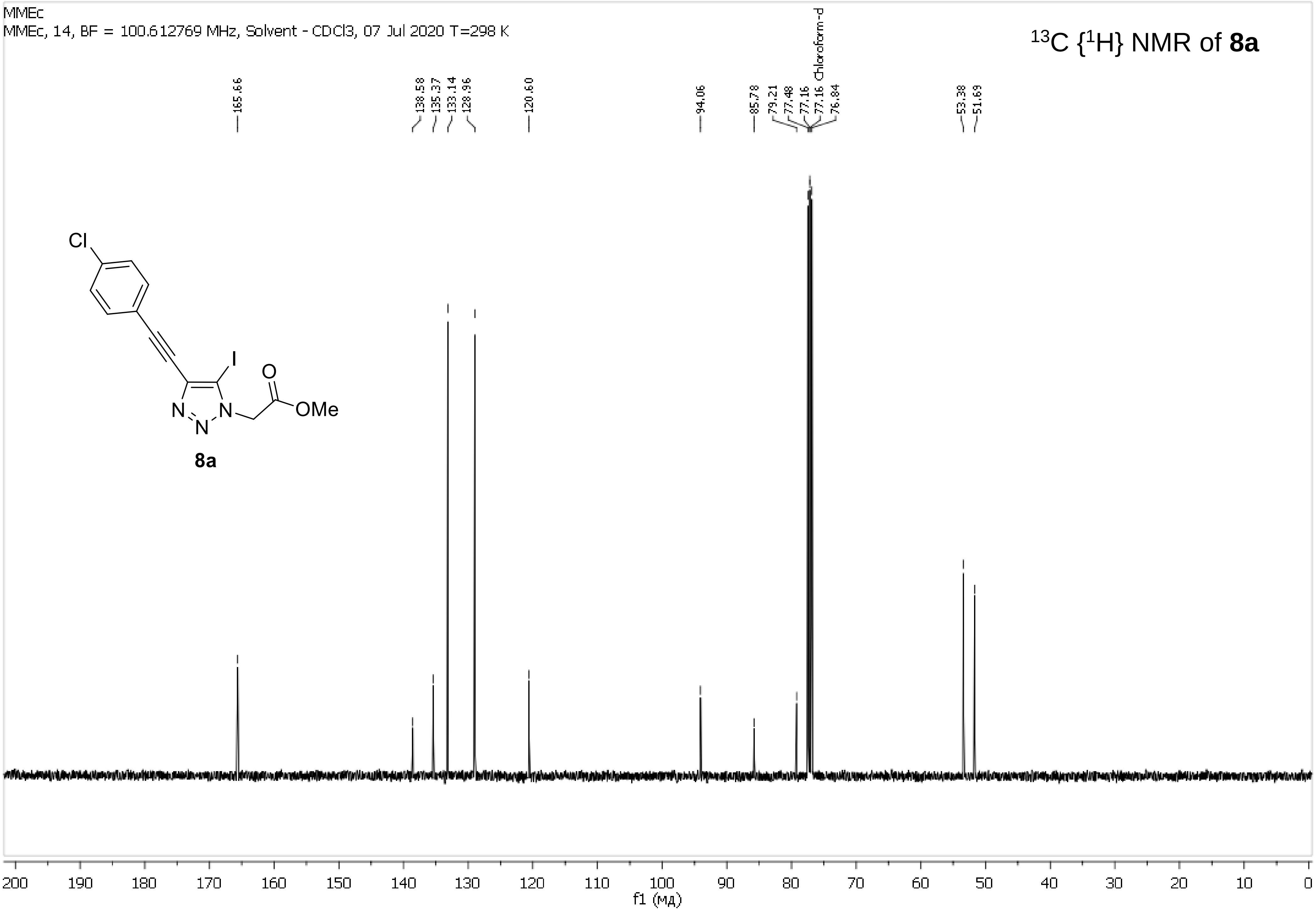
MMEc
MMEc, 14, BF = 100.612769 MHz, Solvent - CDCl3, 07 Jul 2020 T=298 K
13C {1H} NMR of 8a
165.66
138.58
135.37
133.14
128.96
120.60
94.06
85.78
79.21
77.48
77.16
77.16 Chloroform-d
76.84
53.38
51.69
Cl
I
O
OMe
N
N
N
8a
200 190 180 170 160 150 140 130 120 110 100 90 80 70 60 50 40 30 20 10 0
f1 (мд)

MMEd
MMEd, 14, BF = 100.612769 MHz, Solvent - CDCl3, 07 Jul 2020 T=298 K

^{13}C dept NMR of **8a**

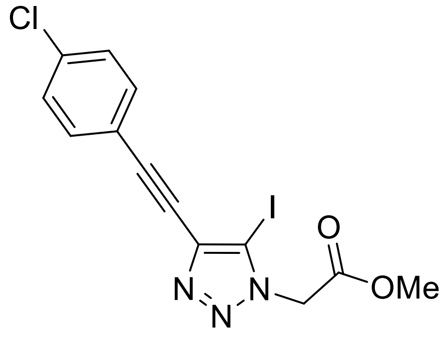

8a

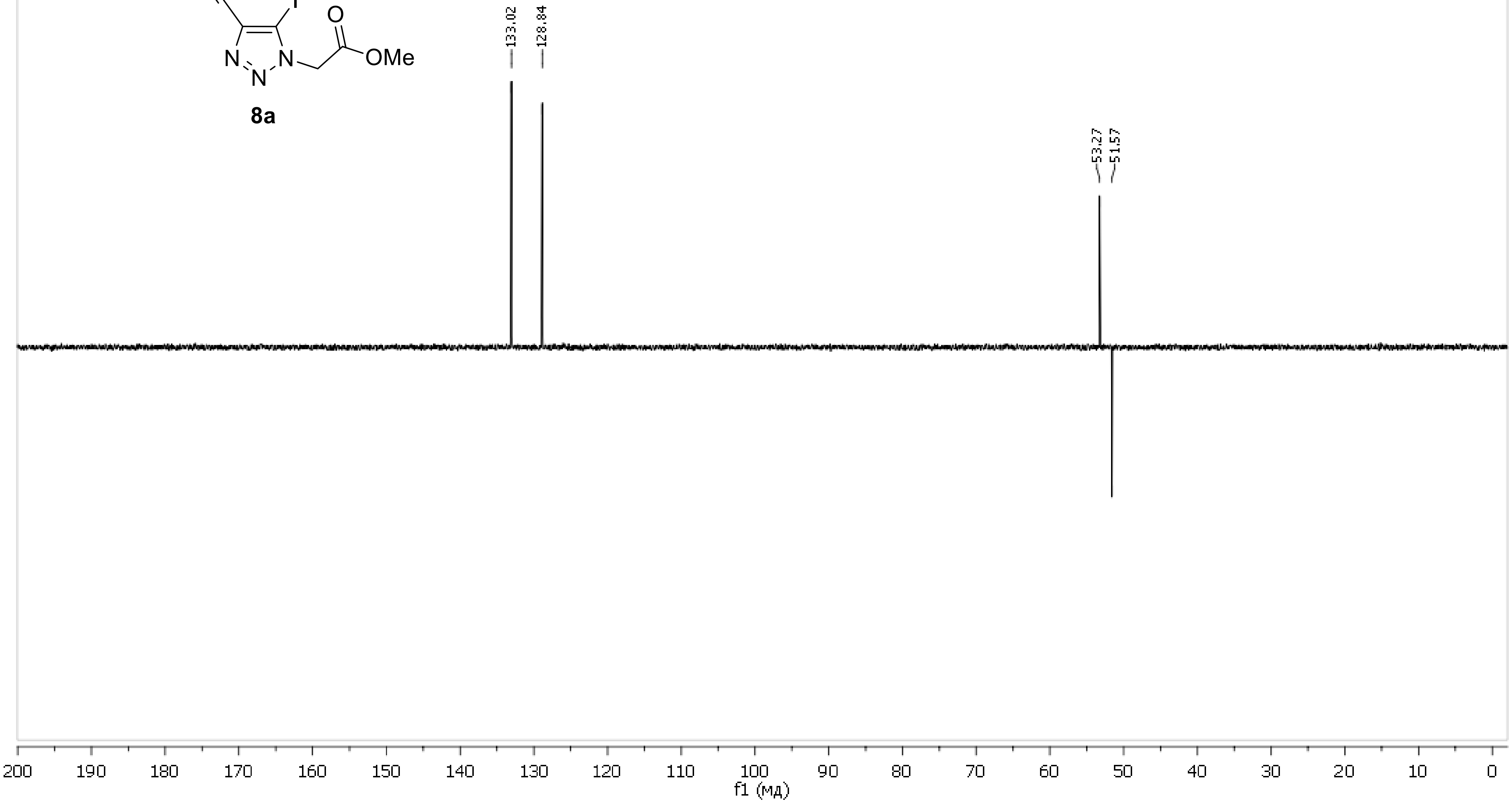

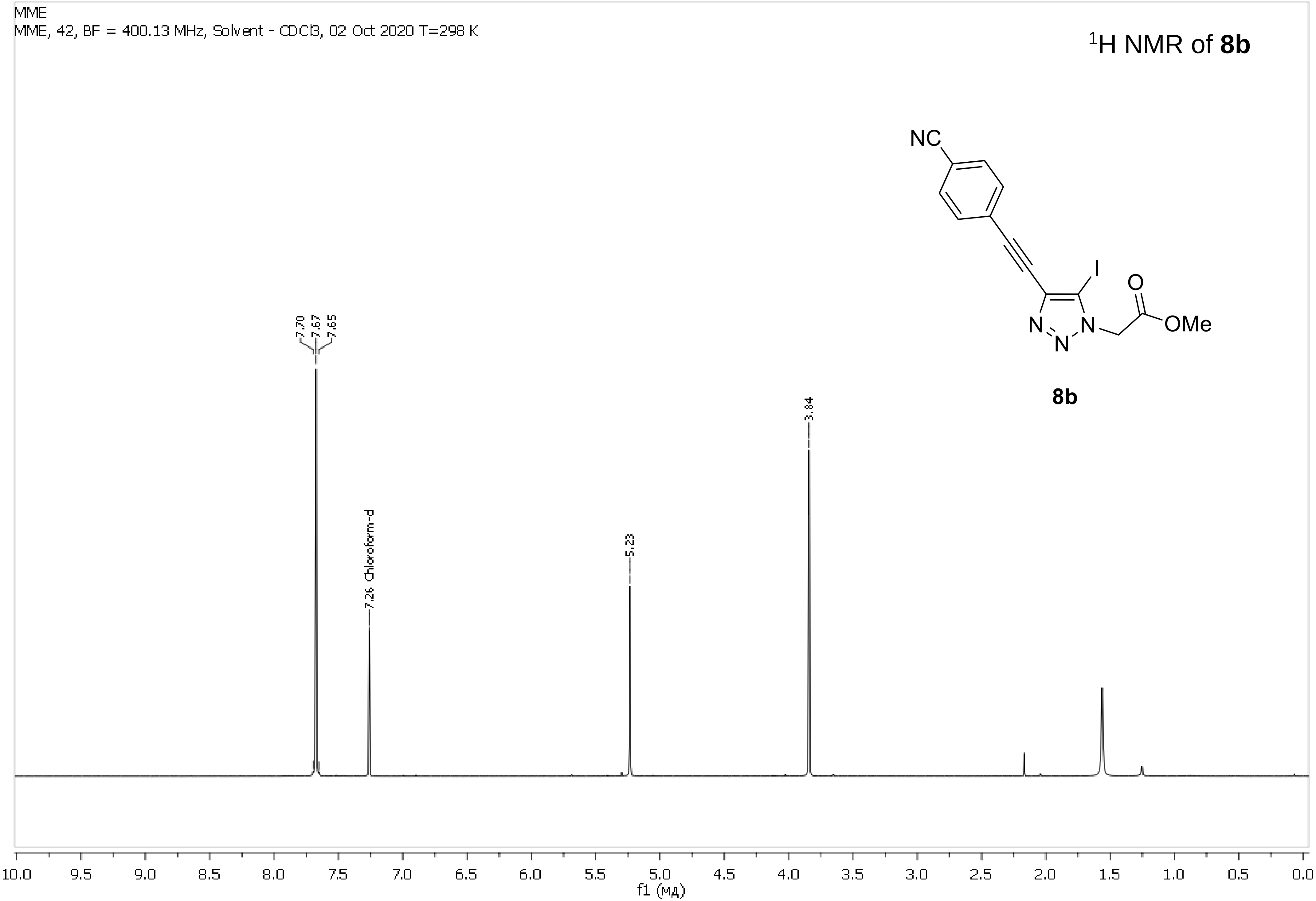
MME
MME, 42, BF = 400.13 MHz, Solvent - CDCl3, 02 Oct 2020 T=298 K
1H NMR of 8b
NC
I
O
OMe
N
N
N
8b
7.70
7.67
7.65
7.26 Chloroform-d
5.23
3.84
10.0
9.5
9.0
8.5
8.0
7.5
7.0
6.5
6.0
5.5
5.0
4.5
4.0
3.5
3.0
2.5
2.0
1.5
1.0
0.5
0.0
f1 (мд)

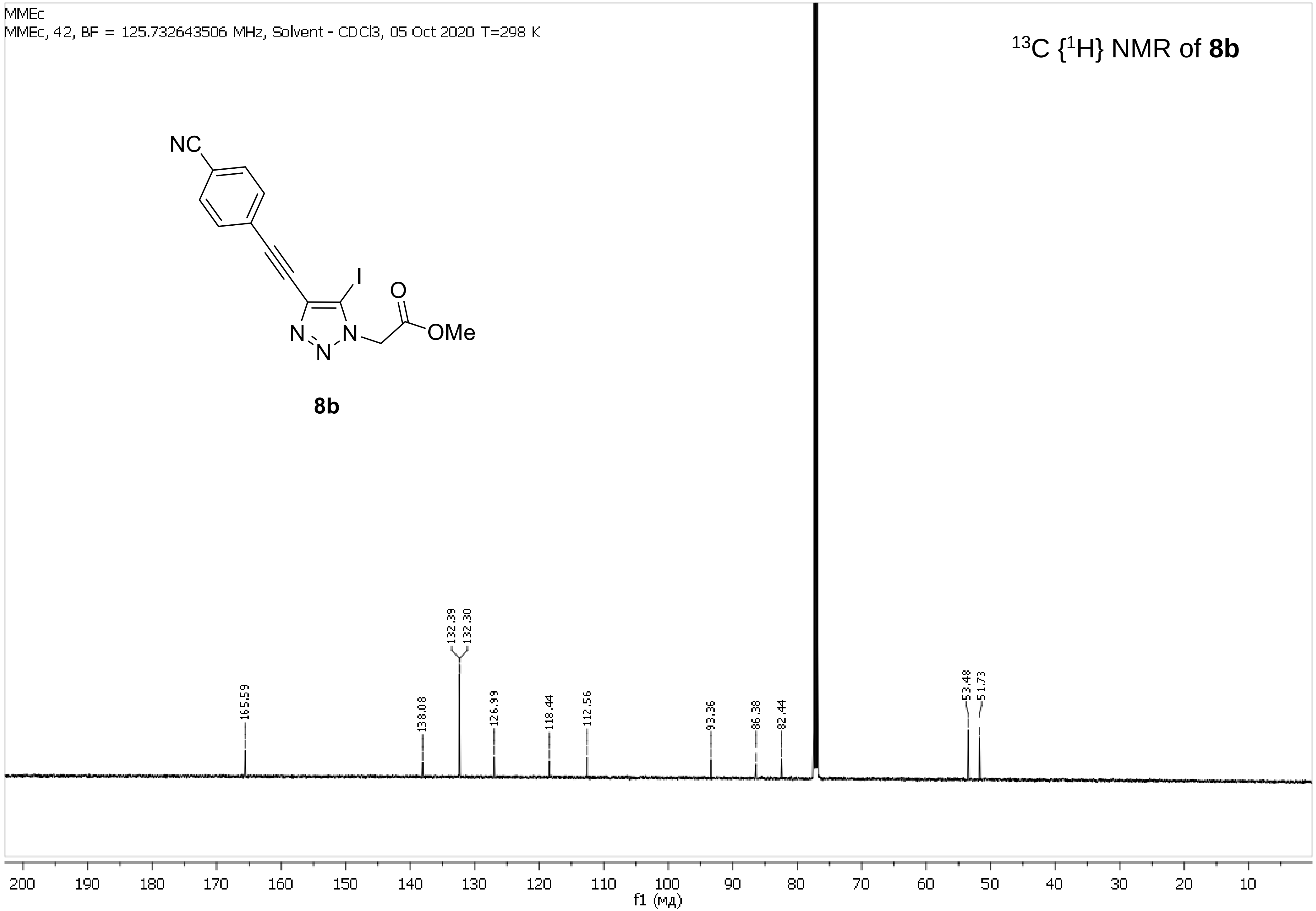
MMEc
MMEc, 42, BF = 125.732643506 MHz, Solvent - CDCl3, 05 Oct 2020 T=298 K
^{13}C {^{1}H} NMR of **8b**
NC
I
O
N
N
N
OMe
8b
165.59
138.08
132.39
132.30
126.99
118.44
112.56
93.36
86.38
82.44
53.48
51.73
200
190
180
170
160
150
140
130
120
110
100
90
80
70
60
50
40
30
20
10
f1 (мд)

MMEd
MMEd, 42, BF = 125.732643506 MHz, Solvent - CDCl3, 05 Oct 2020 T=297 K

^{13}C dept NMR of **8b**

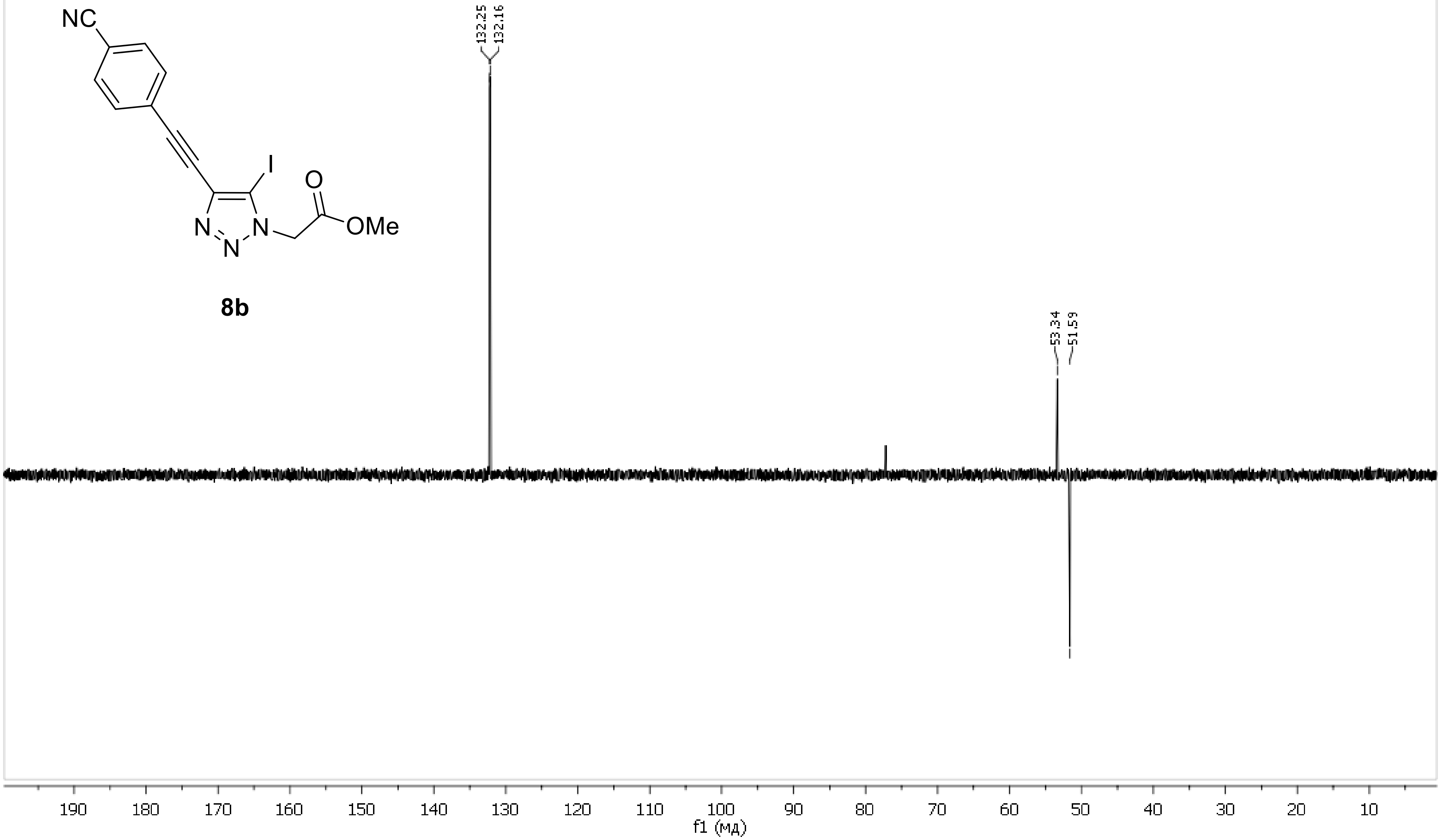

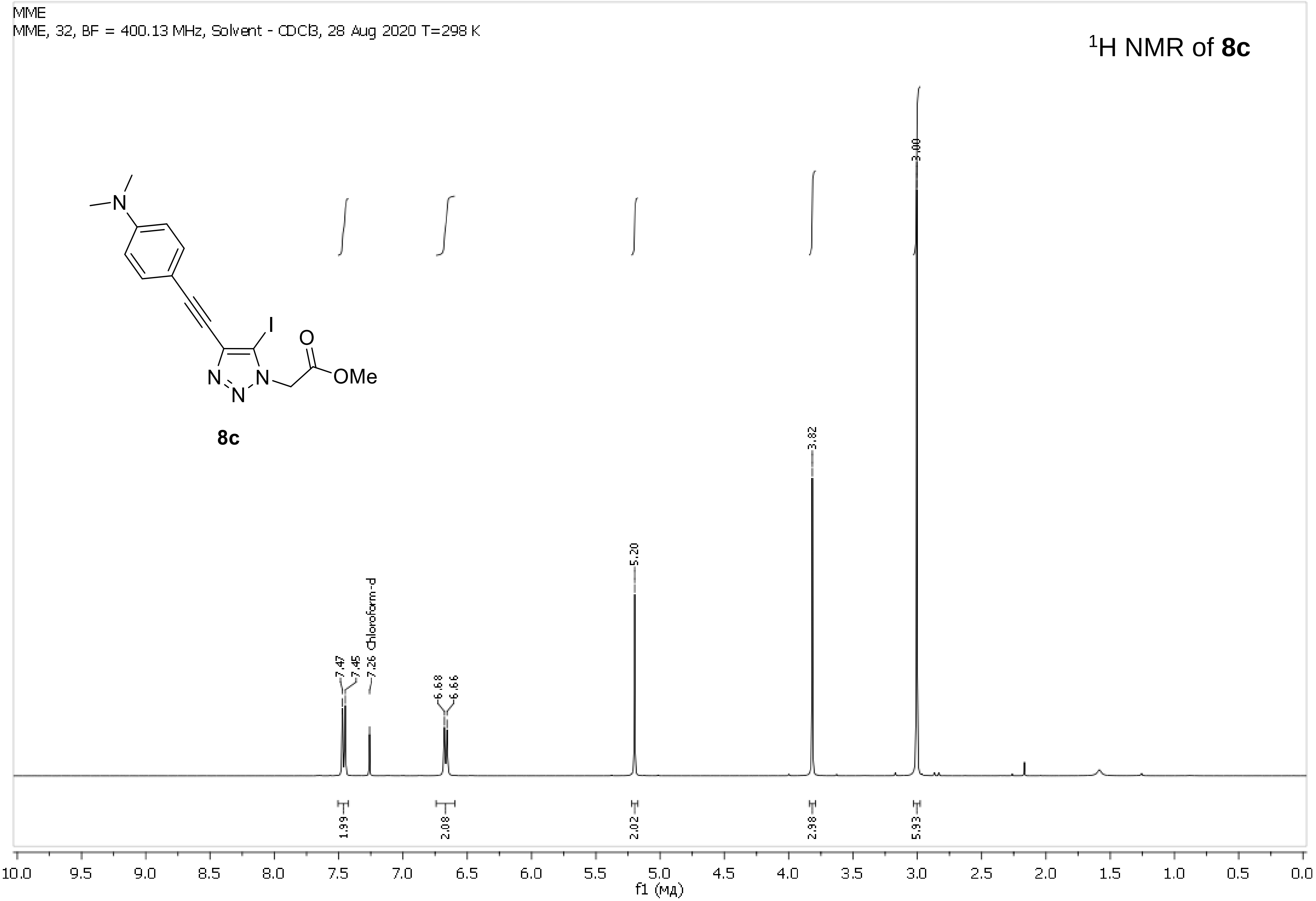
MME
MME, 32, BF = 400.13 MHz, Solvent - CDCl3, 28 Aug 2020 T=298 K
1H NMR of 8c
N
I
O
N
N
N
OMe
8c
7.47
7.45
7.26 Chloroform-d
6.68
6.66
5.20
3.82
3.00
1.99
2.08
2.02
2.98
5.93
10.0 9.5 9.0 8.5 8.0 7.5 7.0 6.5 6.0 5.5 5.0 4.5 4.0 3.5 3.0 2.5 2.0 1.5 1.0 0.5 0.0
f1 (мд)

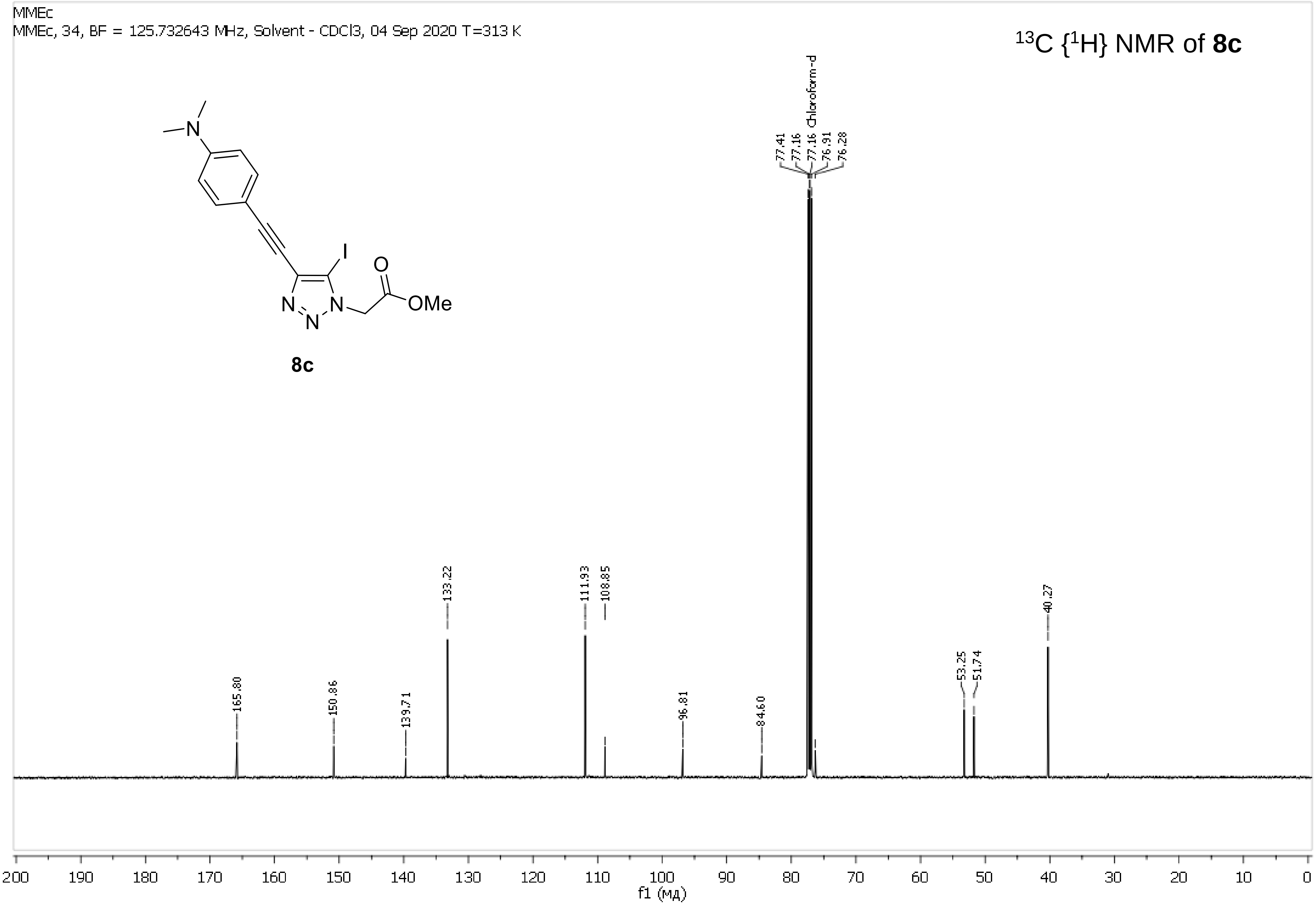

MMEc
MMEc, 34, BF = 125.732643 MHz, Solvent - CDCl3, 04 Sep 2020 T=313 K
^{13}C {^{1}H} NMR of **8c**
N
I
O
N
N
N
OMe
8c
Chloroform-d
77.41
77.16
77.16
76.91
76.28
165.80
150.86
139.71
133.22
111.93
108.85
96.81
84.60
53.25
51.74
40.27
200 190 180 170 160 150 140 130 120 110 100 90 80 70 60 50 40 30 20 10 0
f1 (мд)

MMEd
MMEd, 34, BF = 125.732643 MHz, Solvent - CDCl3, 04 Sep 2020 T=313 K

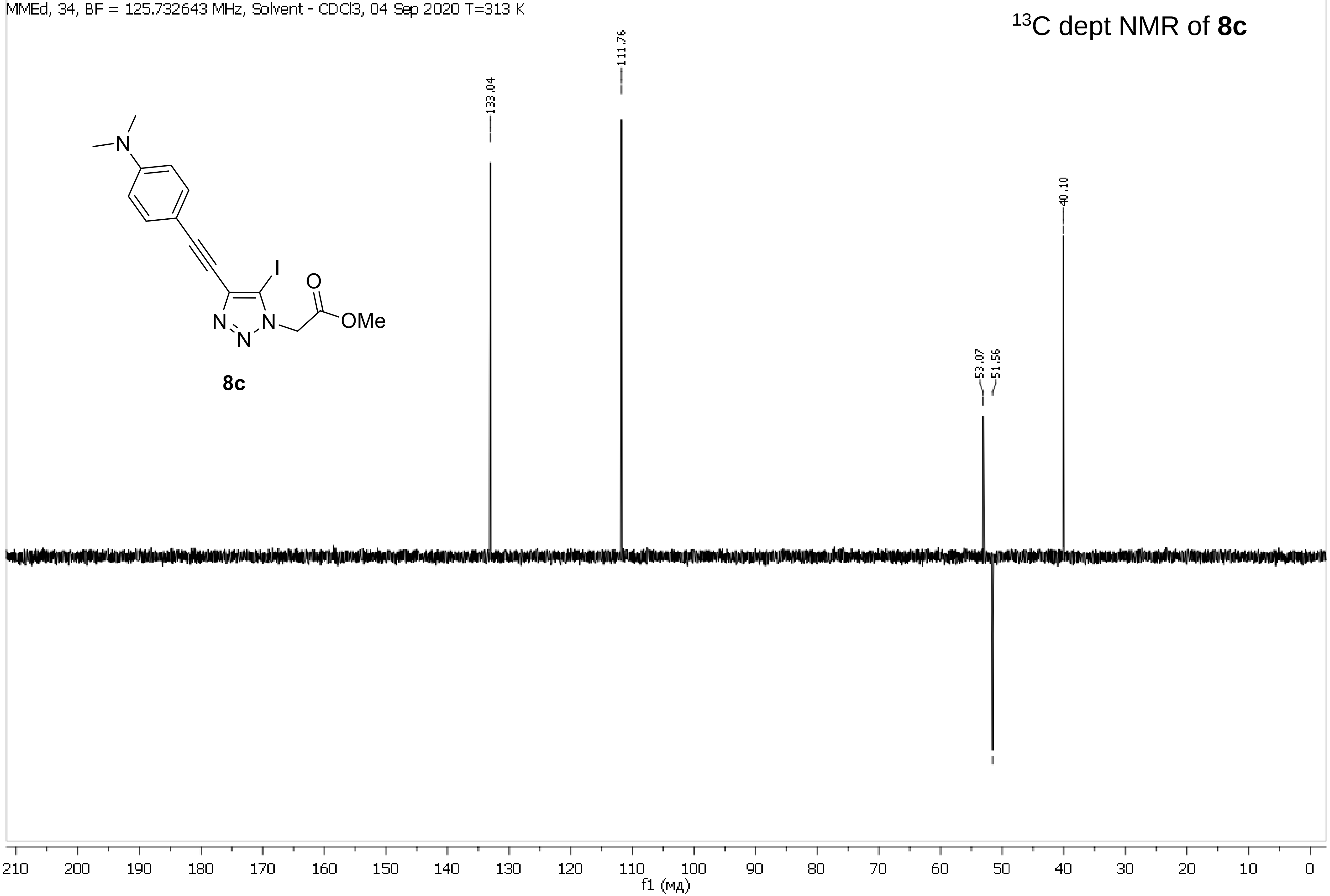

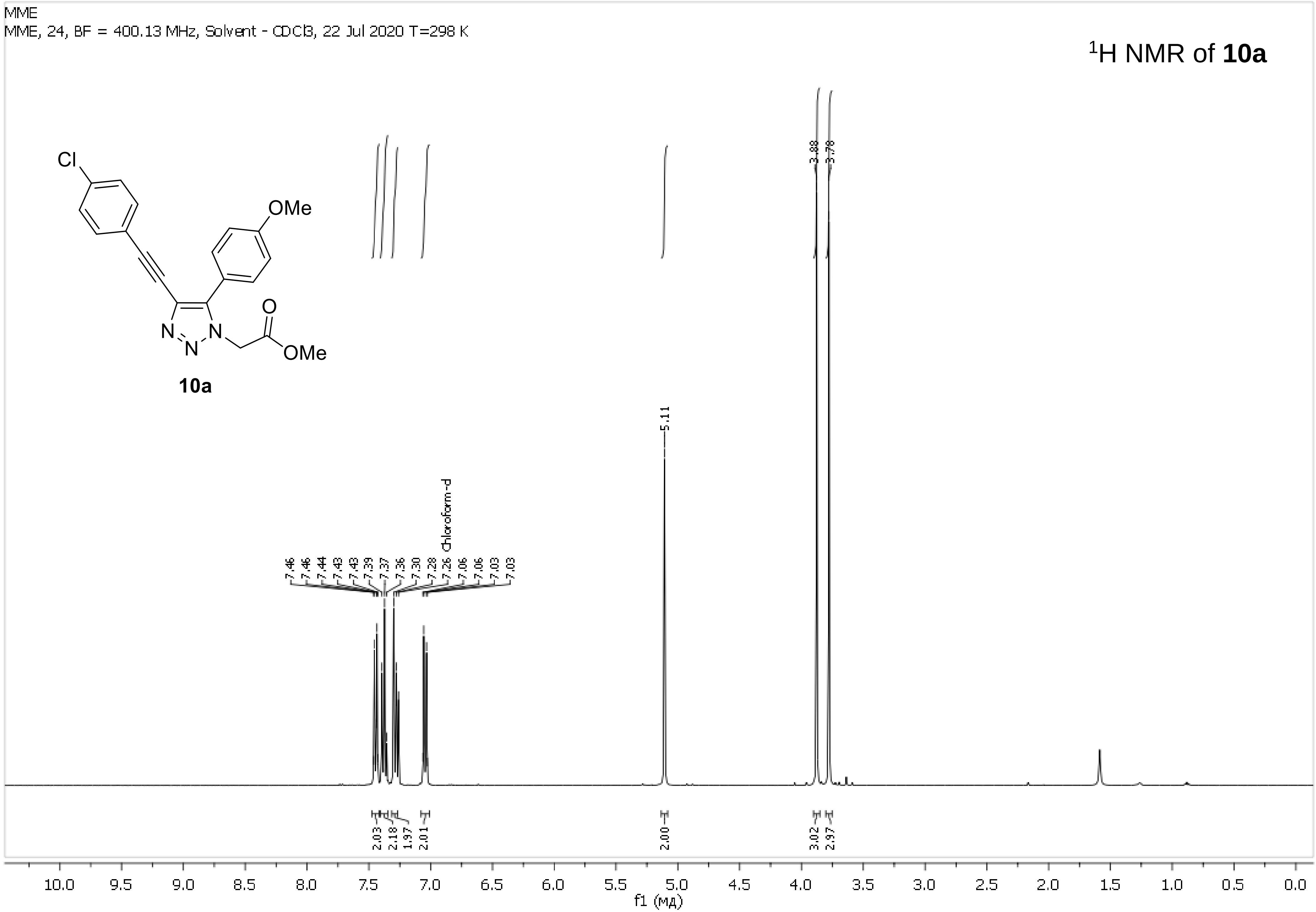
MME
MME, 24, BF = 400.13 MHz, Solvent - CDCl3, 22 Jul 2020 T=298 K
1H NMR of 10a
Cl
OMe
O
N
N
N
OMe
10a
7.46 7.46 7.44 7.43 7.43 7.39 7.37 7.36 7.30 7.28 7.26 Chloroform-d 7.06 7.06 7.03 7.03
5.11
3.88
3.78
2.03 2.18 1.97 2.01
2.00
3.02 2.97
10.0 9.5 9.0 8.5 8.0 7.5 7.0 6.5 6.0 5.5 5.0 4.5 4.0 3.5 3.0 2.5 2.0 1.5 1.0 0.5 0.0
f1 (мд)

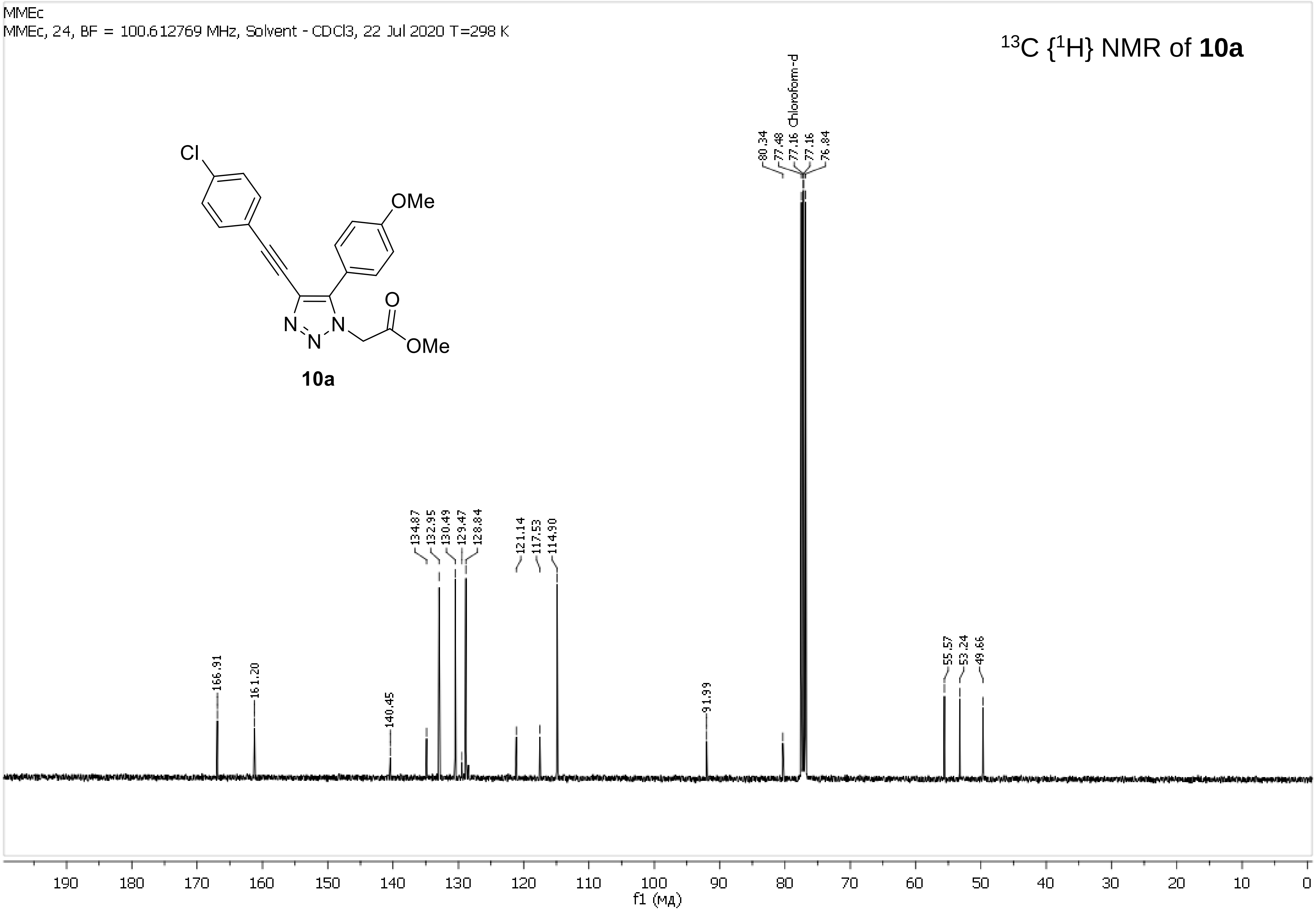
MMEc
MMEc, 24, BF = 100.612769 MHz, Solvent - CDCl3, 22 Jul 2020 T=298 K
^{13}C {^{1}H} NMR of **10a**
Cl
OMe
O
N
N
N
OMe
10a
Chloroform-d
80.34
77.48
77.16
77.16
76.84
134.87
132.95
130.49
129.47
128.84
121.14
117.53
114.90
166.91
161.20
140.45
91.99
55.57
53.24
49.66
190 180 170 160 150 140 130 120 110 100 90 80 70 60 50 40 30 20 10 0
f1 (мд)

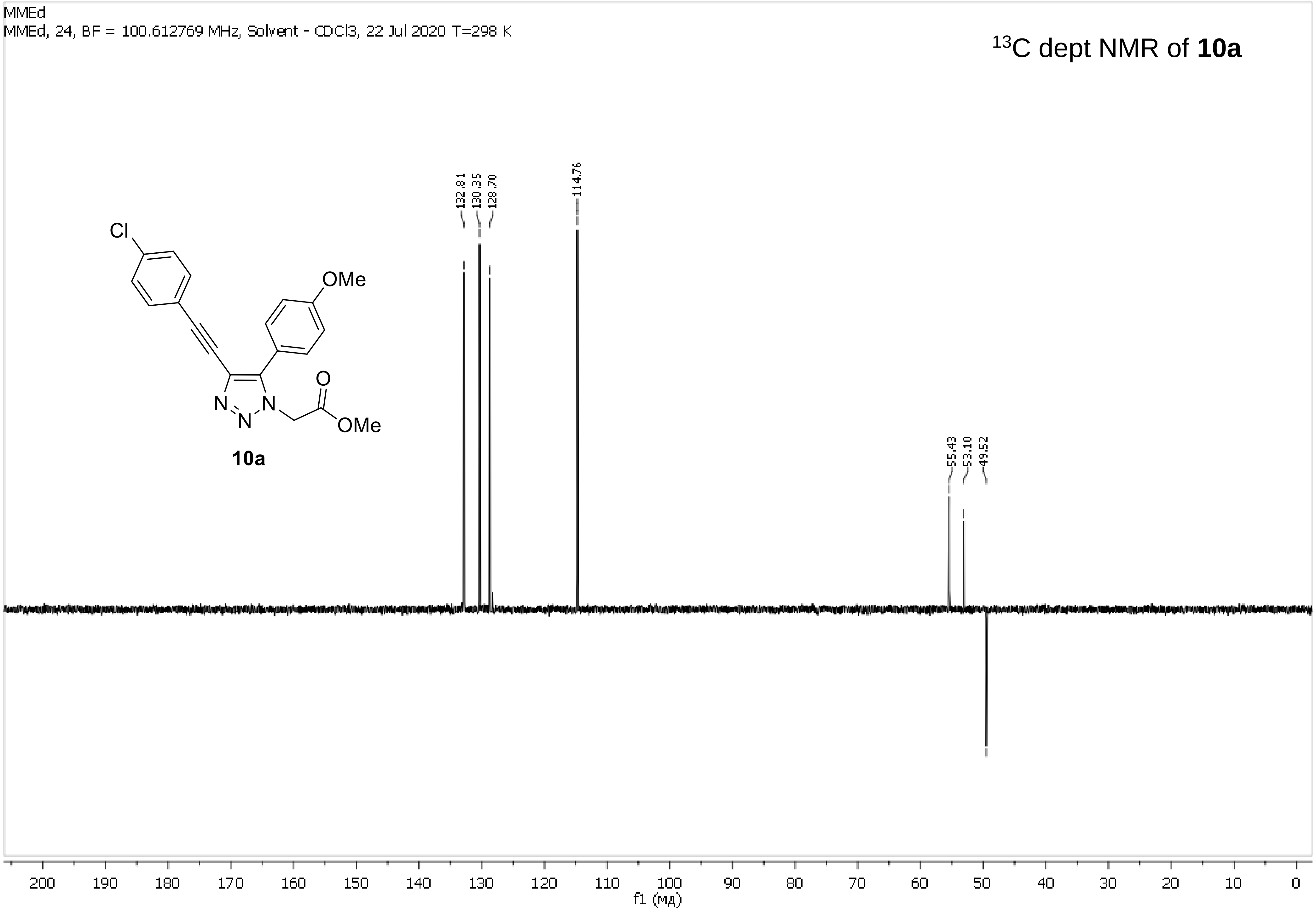
MMEd
MMEd, 24, BF = 100.612769 MHz, Solvent - CDCl3, 22 Jul 2020 T=298 K
13C dept NMR of 10a
132.81
130.35
128.70
114.76
55.43
53.10
49.52
Cl
OMe
O
N
N
N
OMe
10a
200 190 180 170 160 150 140 130 120 110 100 90 80 70 60 50 40 30 20 10 0
f1 (мд)

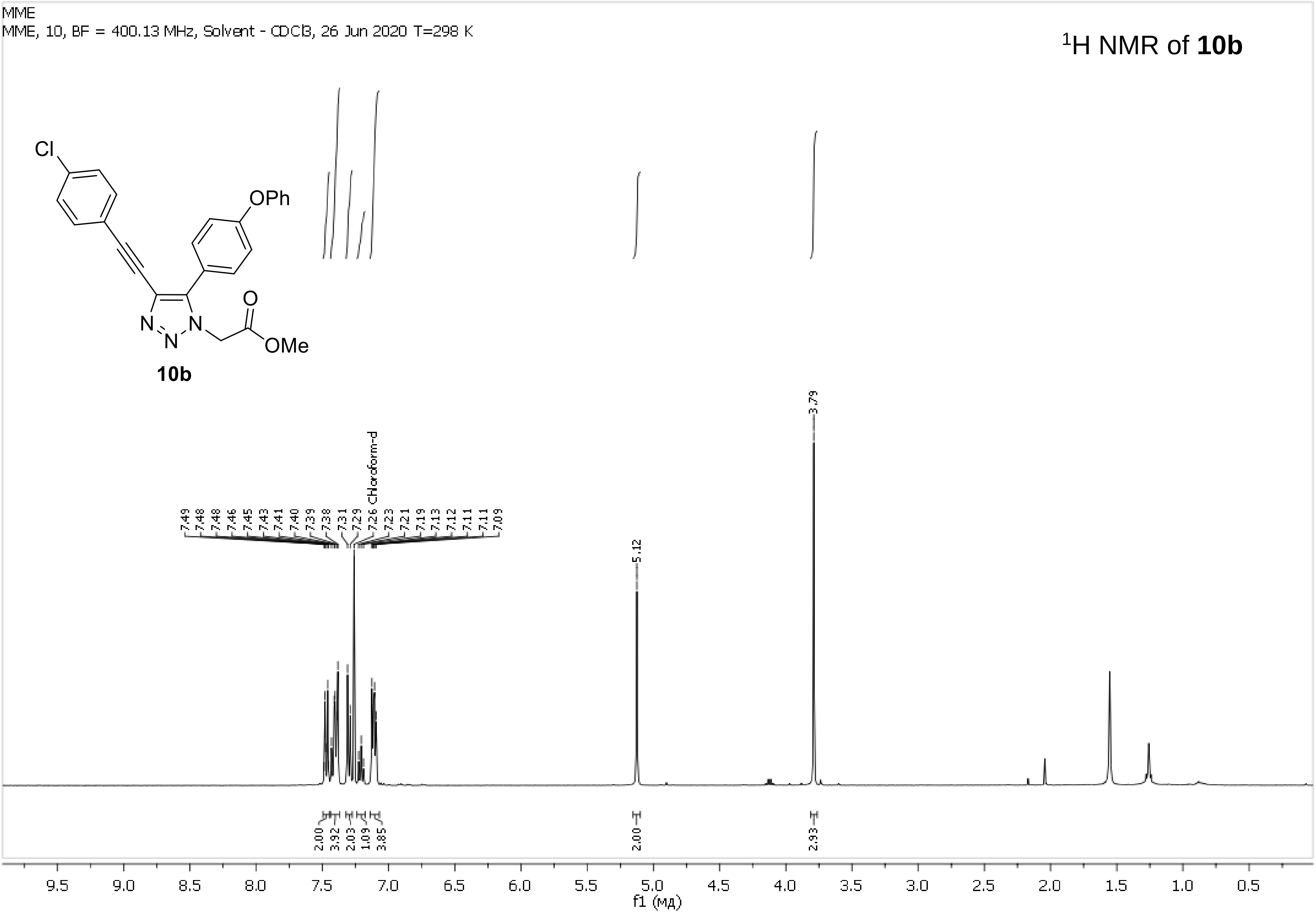
MME
MME, 10, BF = 400.13 MHz, Solvent - CDCl3, 26 Jun 2020 T=298 K
1H NMR of 10b
Cl
OPh
O
N
N
N
OMe
10b
7.49 7.48 7.48 7.46 7.45 7.43 7.43 7.41 7.40 7.39 7.38 7.31 7.29 7.26 7.23 7.21 7.19 7.13 7.12 7.11 7.11 7.09
Chloroform-d
5.12
3.79
2.00 3.92 2.03 1.09 3.85
2.00
2.93
9.5 9.0 8.5 8.0 7.5 7.0 6.5 6.0 5.5 5.0 4.5 4.0 3.5 3.0 2.5 2.0 1.5 1.0 0.5
f1 (мд)

MMEc
MMEc, 21, BF = 100.612769 MHz, Solvent - CDCl3, 22 Jul 2020 T=298 K

$^{13}C\{^{1}H\}$ NMR of **10b**

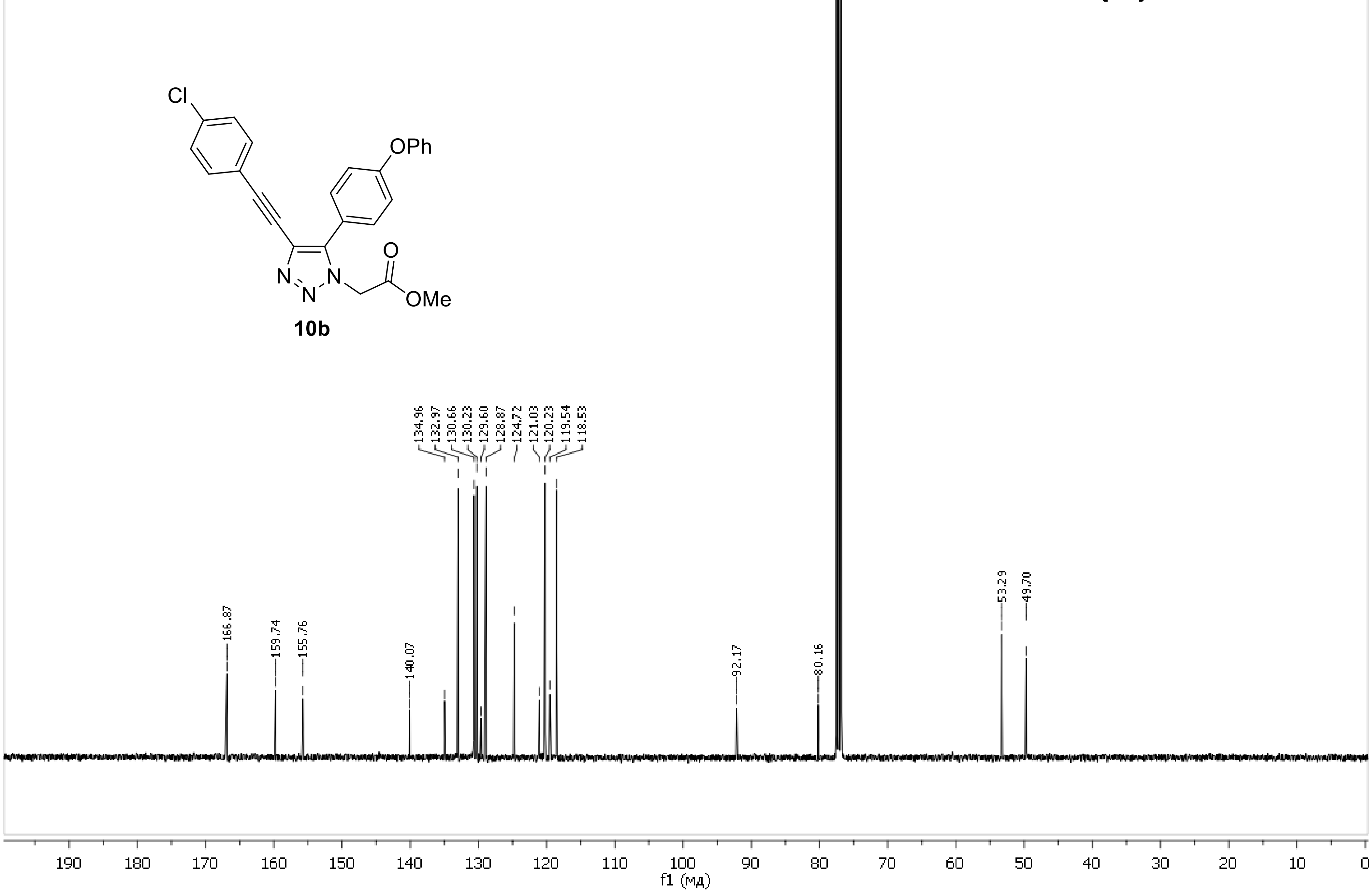

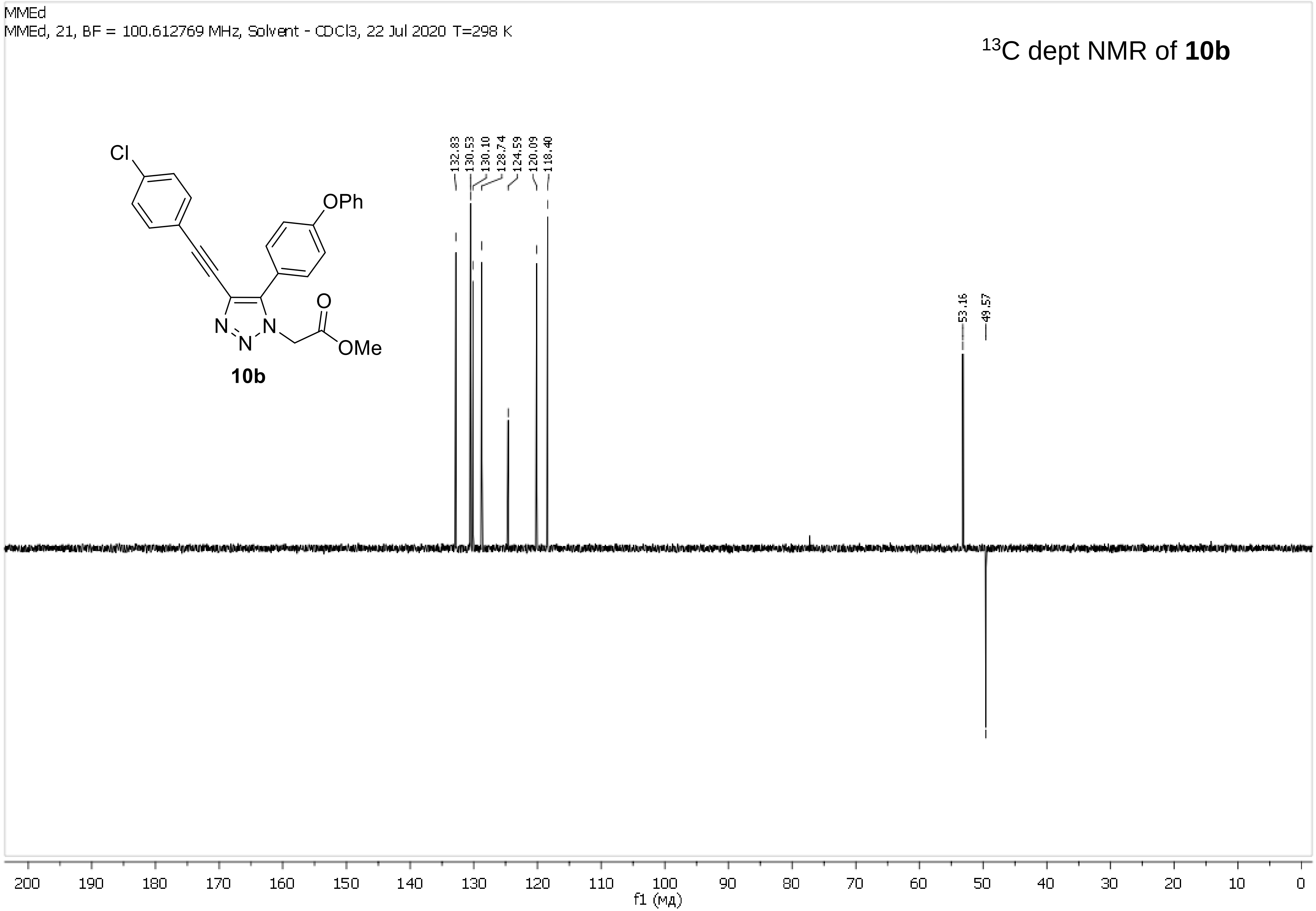

MMEd
MMEd, 21, BF = 100.612769 MHz, Solvent - CDCl3, 22 Jul 2020 T=298 K
13C dept NMR of 10b
Cl
OPh
O
N
N
N
OMe
10b
132.83
130.53
130.10
128.74
124.59
120.09
118.40
53.16
49.57
200
190
180
170
160
150
140
130
120
110
100
90
80
70
60
50
40
30
20
10
0
f1 (мд)

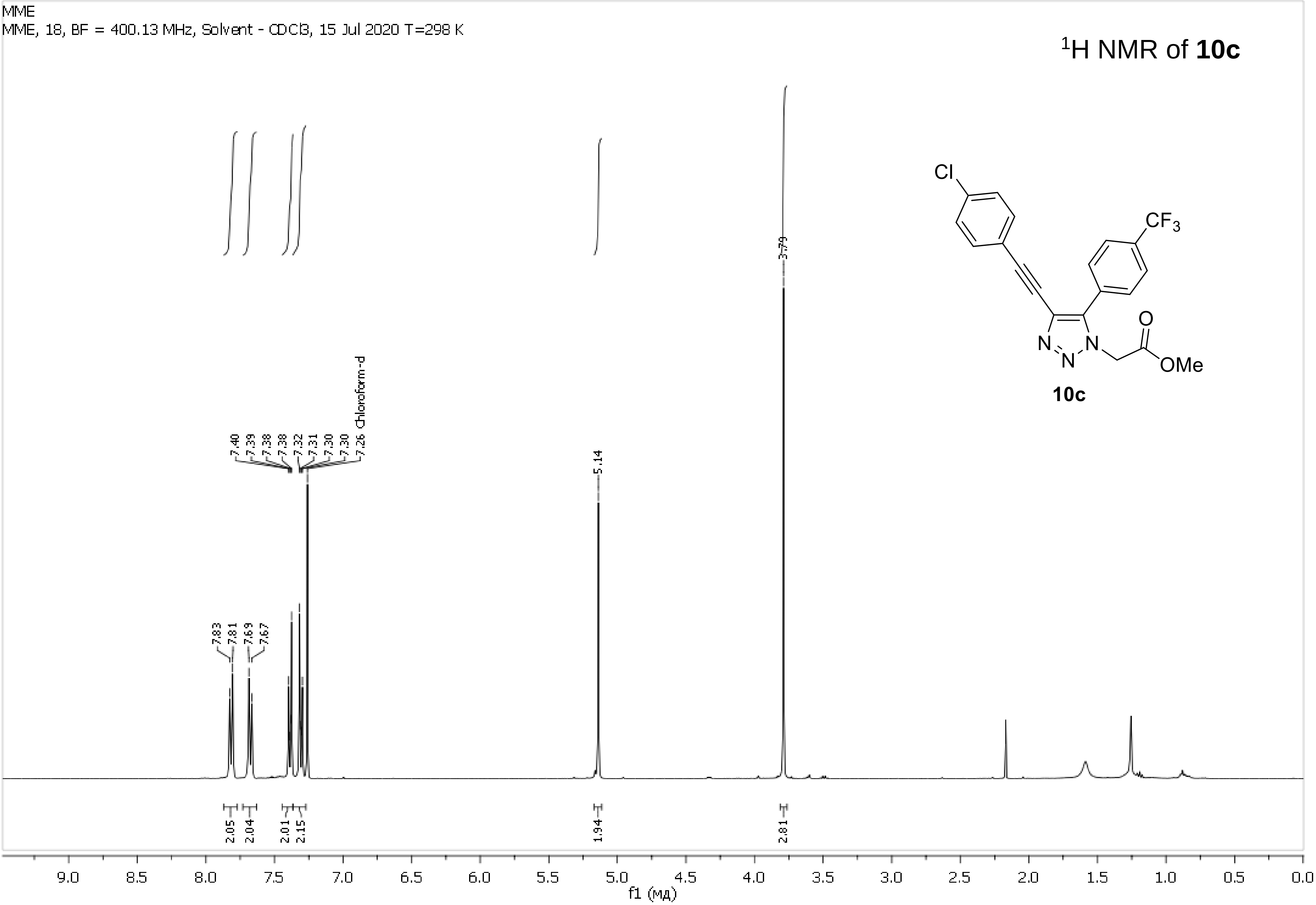
MME
MME, 18, BF = 400.13 MHz, Solvent - CDCl3, 15 Jul 2020 T=298 K
1H NMR of 10c
Cl
CF3
O
N
N
N
OMe
10c
7.83
7.81
7.69
7.67
7.40
7.39
7.38
7.38
7.32
7.31
7.30
7.30
7.26 Chloroform-d
5.14
3.79
2.05
2.04
2.01
2.15
1.94
2.81
9.0 8.5 8.0 7.5 7.0 6.5 6.0 5.5 5.0 4.5 4.0 3.5 3.0 2.5 2.0 1.5 1.0 0.5 0.0
f1 (мд)

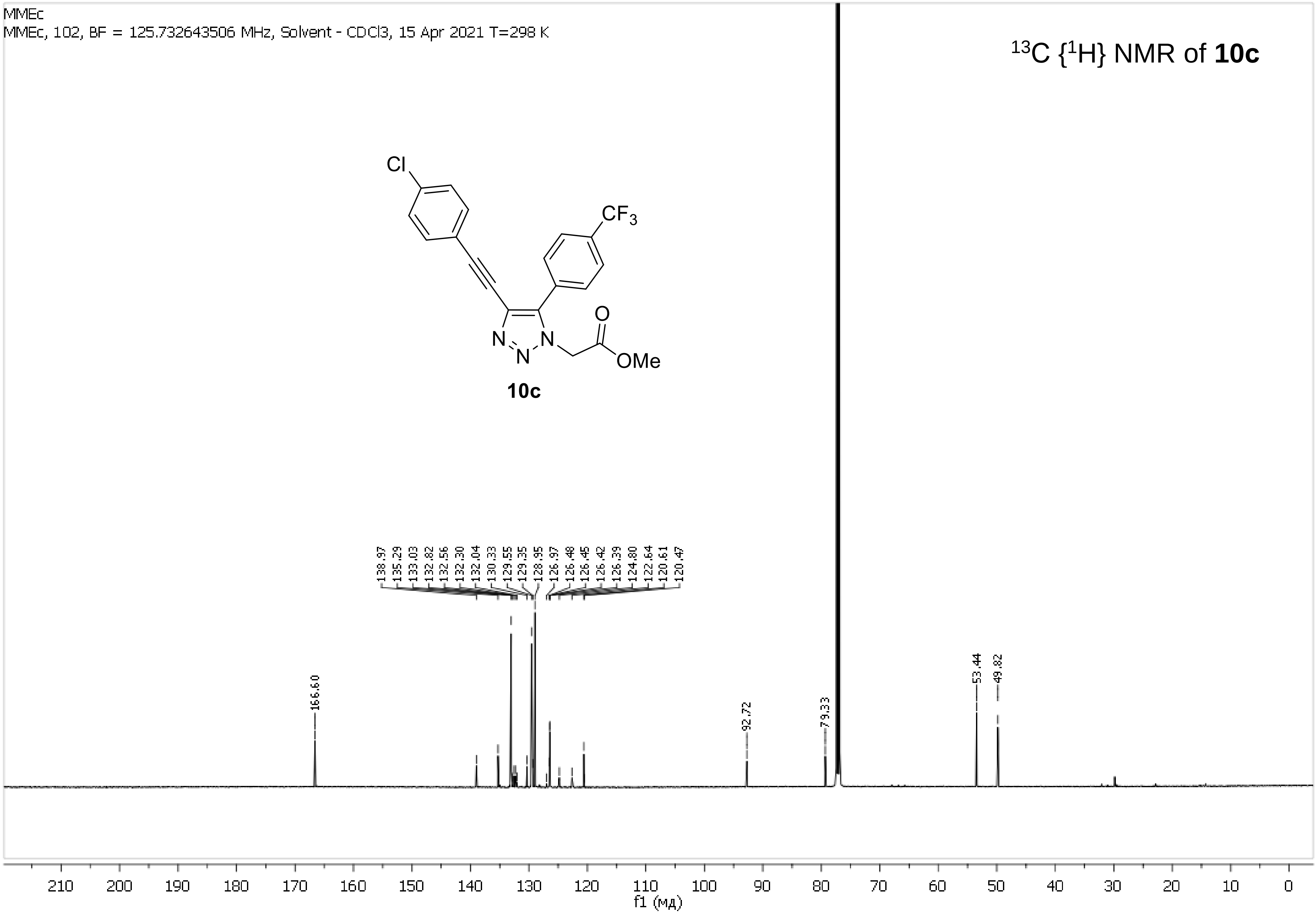
MMEc
MMEc, 102, BF = 125.732643506 MHz, Solvent - CDCl3, 15 Apr 2021 T=298 K
$^{13}C\ \{^{1}H\}$ NMR of **10c**
Cl
CF_3
O
N
N
N
OMe
10c
166.60
138.97
135.29
133.03
132.82
132.56
132.30
132.04
130.33
129.55
129.35
128.95
126.97
126.48
126.45
126.42
126.39
124.80
122.64
120.61
120.47
92.72
79.33
53.44
49.82
210 200 190 180 170 160 150 140 130 120 110 100 90 80 70 60 50 40 30 20 10 0
f1 (мд)

MMEd
MMEd, 18, BF = 100.612769 MHz, Solvent - CDCl3, 16 Jul 2020 T=298 K

^{13}C dept NMR of **10c**

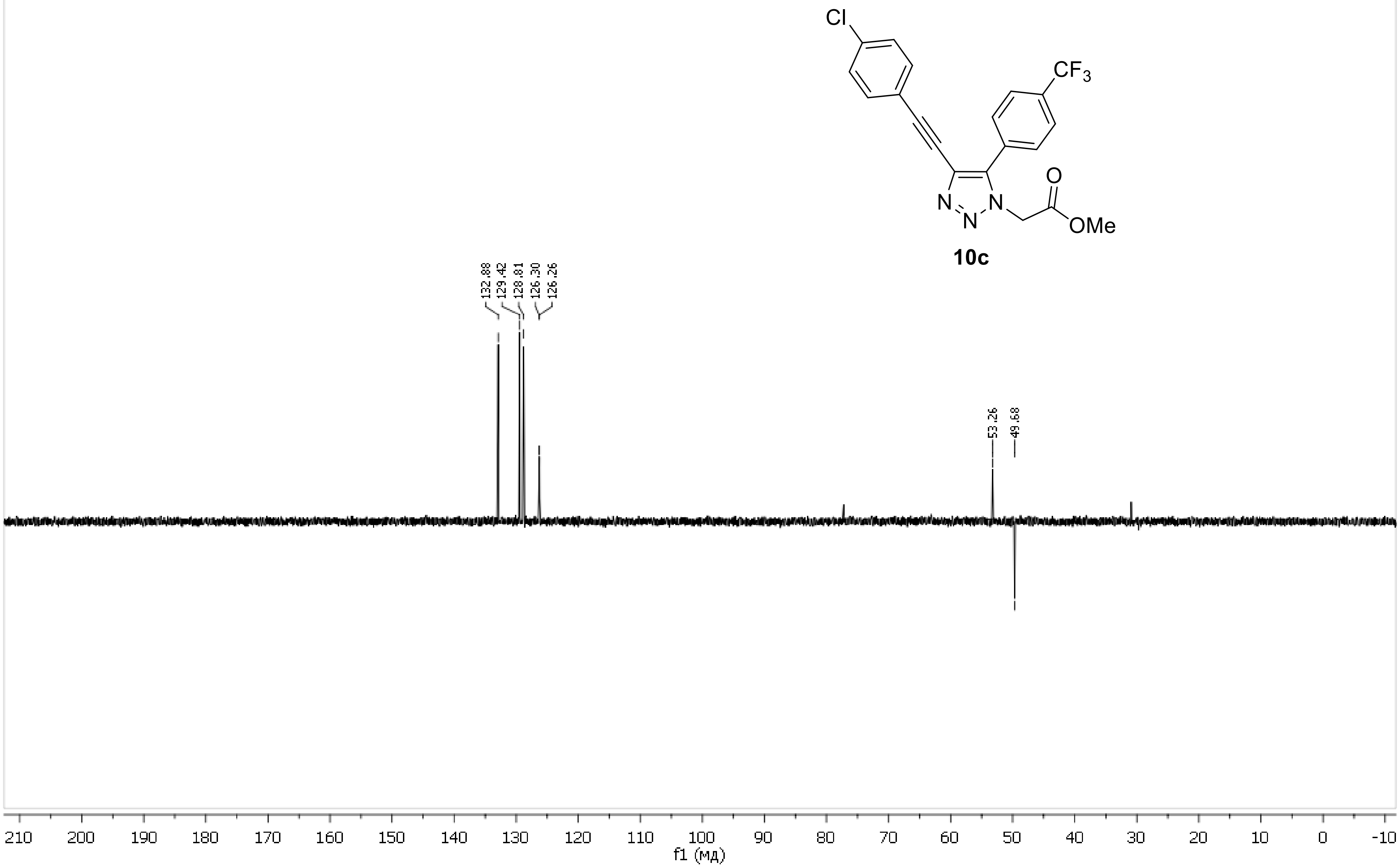

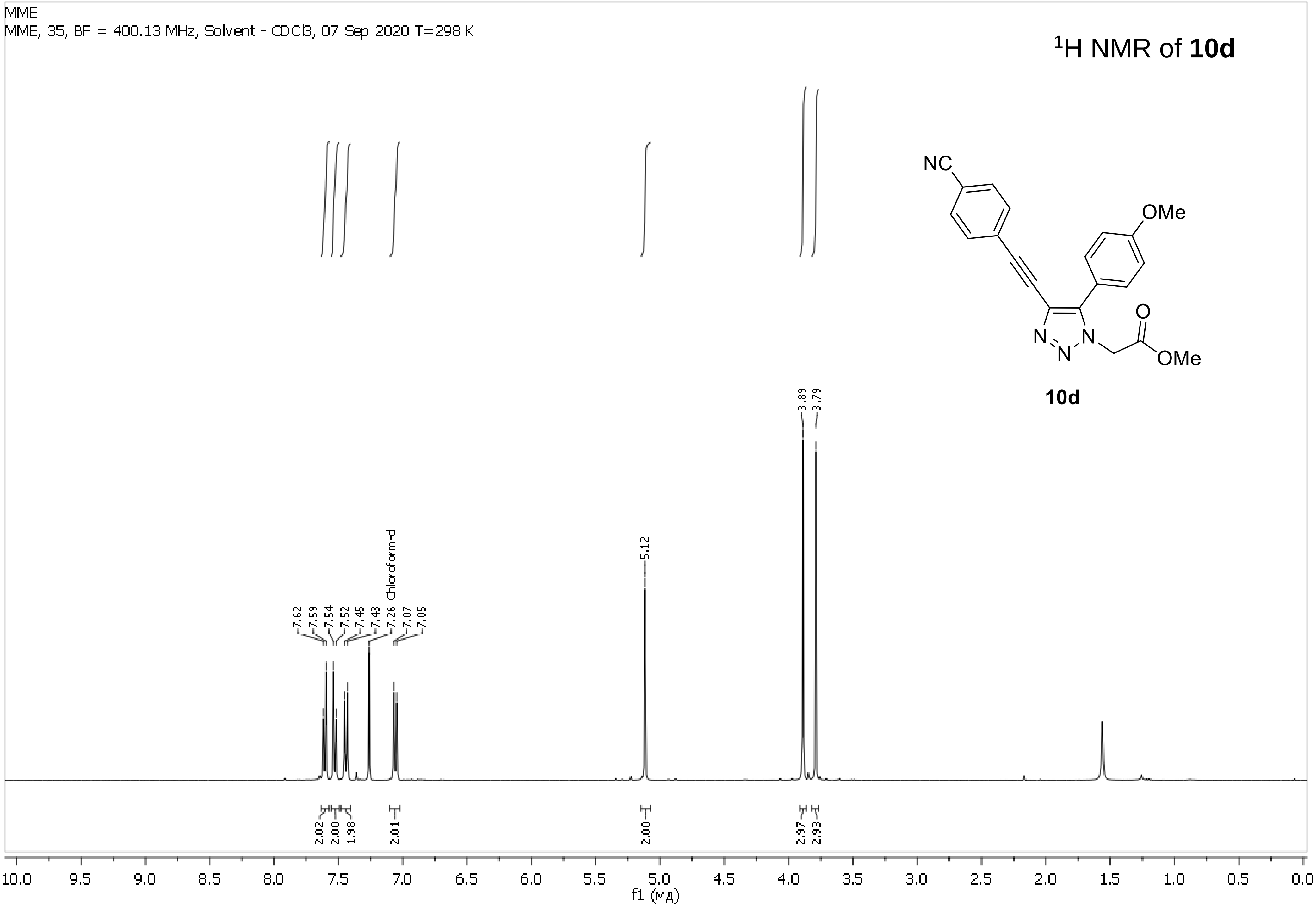
MME
MME, 35, BF = 400.13 MHz, Solvent - CDCl3, 07 Sep 2020 T=298 K
1H NMR of 10d
NC
OMe
O
N
N
N
OMe
10d
7.62
7.59
7.54
7.52
7.45
7.43
7.26 Chloroform-d
7.07
7.05
5.12
3.89
3.79
2.02
2.00
1.98
2.01
2.00
2.97
2.93
10.0
9.5
9.0
8.5
8.0
7.5
7.0
6.5
6.0
5.5
5.0
4.5
4.0
3.5
3.0
2.5
2.0
1.5
1.0
0.5
0.0
f1 (мд)

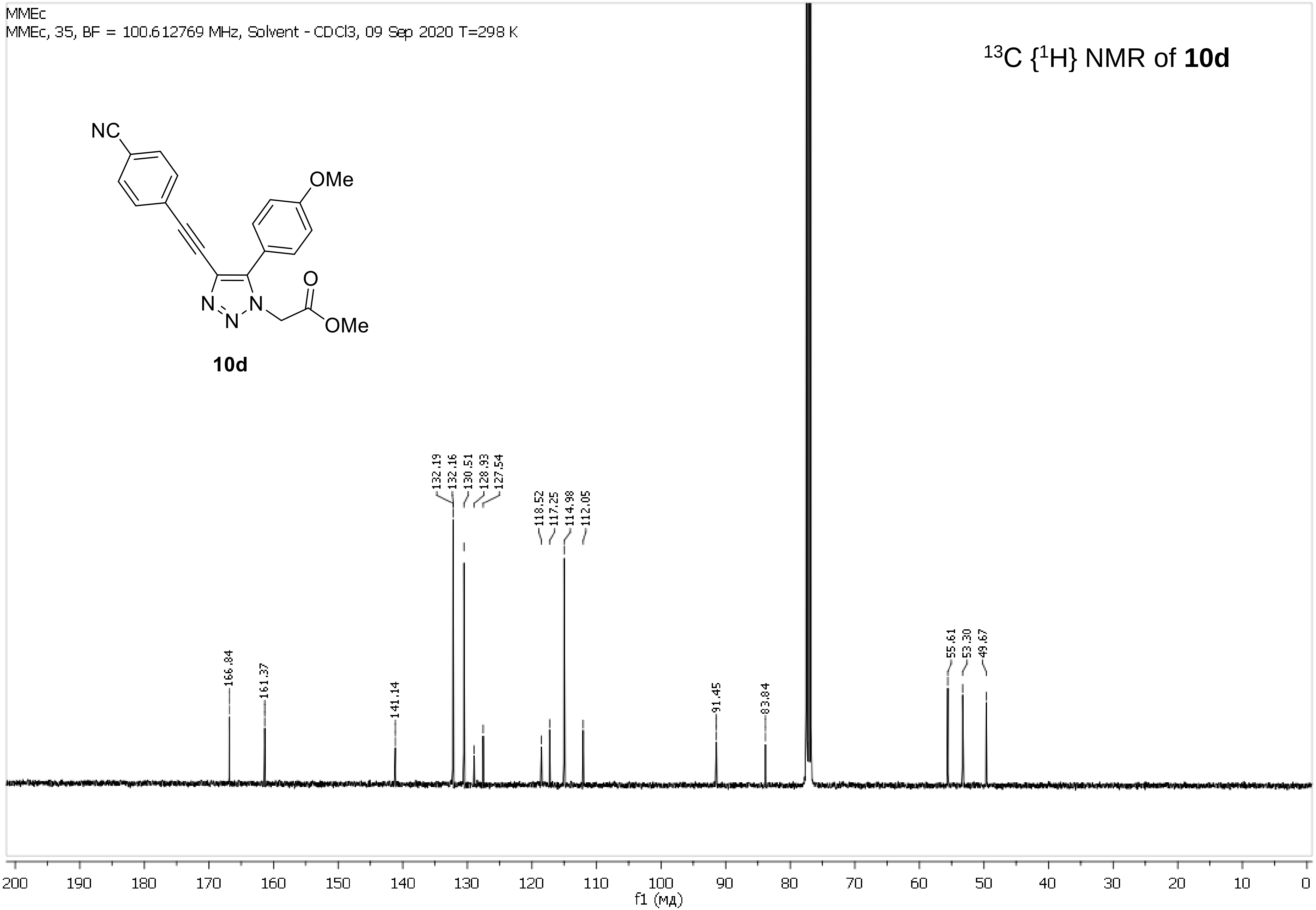
MMEc
MMEc, 35, BF = 100.612769 MHz, Solvent - CDCl3, 09 Sep 2020 T=298 K
13C {1H} NMR of 10d
NC
OMe
O
N
N
N
OMe
10d
166.84
161.37
141.14
132.19
132.16
130.51
128.93
127.54
118.52
117.25
114.98
112.05
91.45
83.84
55.61
53.30
49.67
200 190 180 170 160 150 140 130 120 110 100 90 80 70 60 50 40 30 20 10 0
f1 (мд)

MMEd
MMEd, 35, BF = 100.612769 MHz, Solvent - CDCl3, 09 Sep 2020 T=298 K

^{13}C dept NMR of **10d**

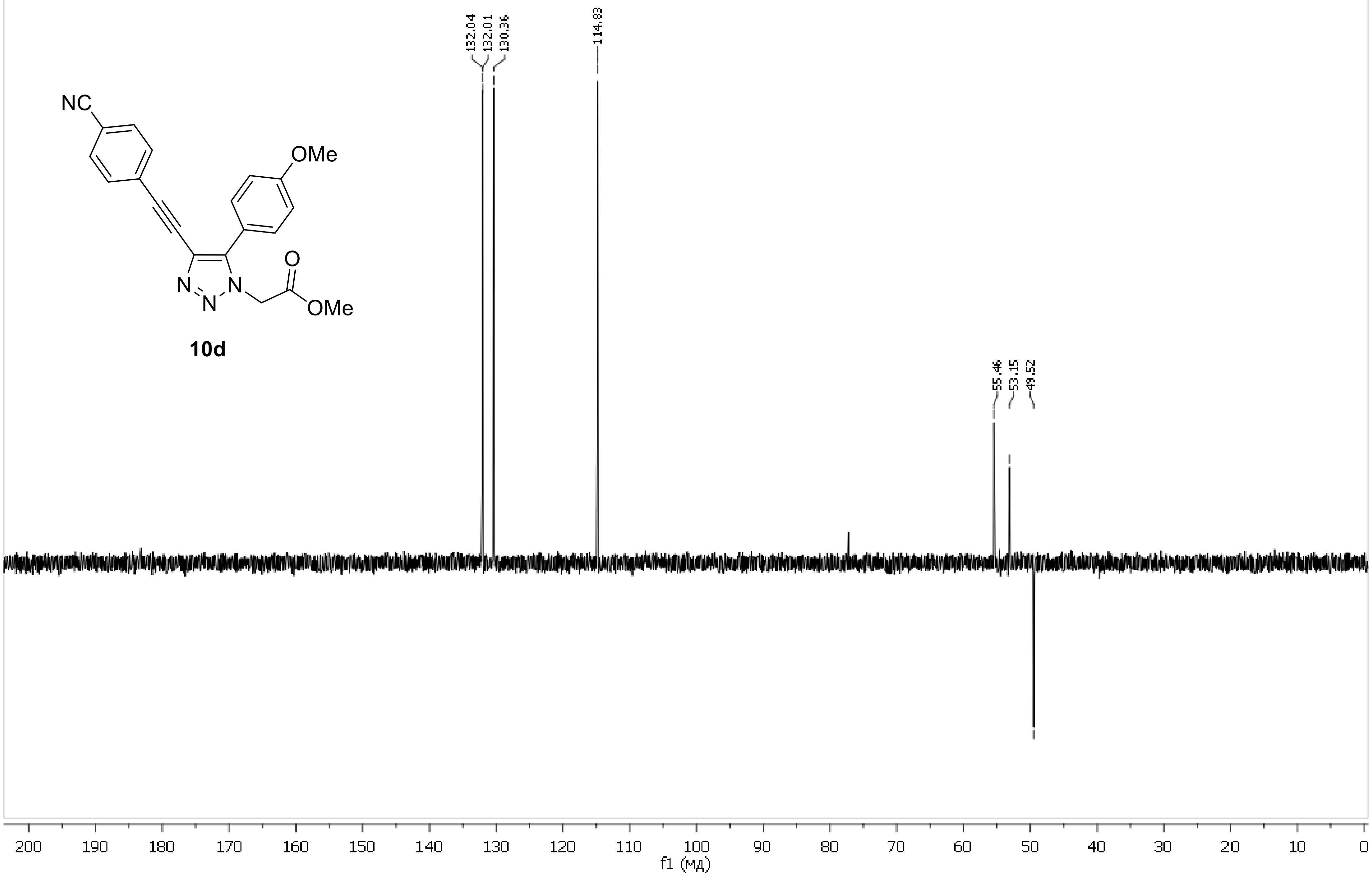

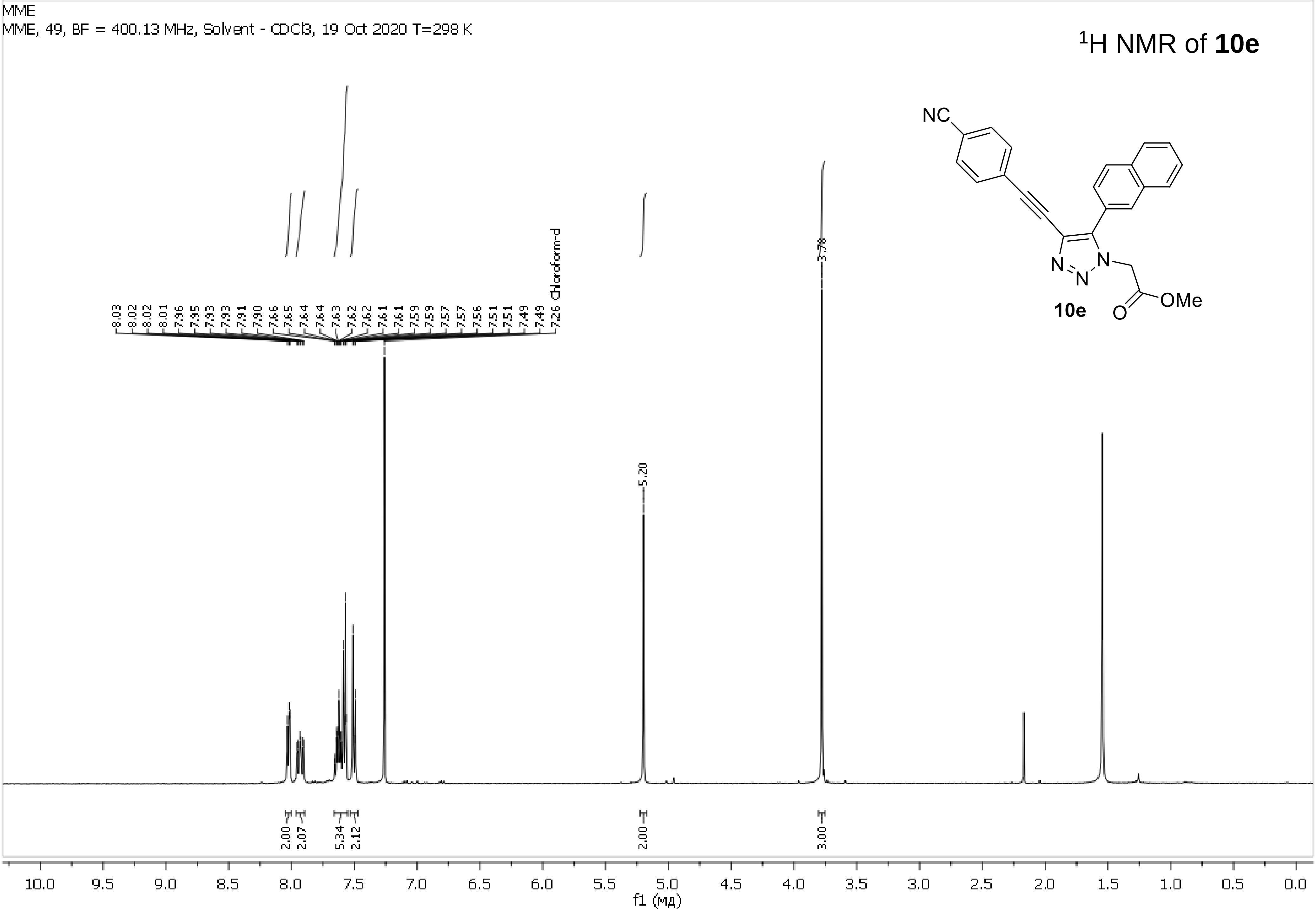
MME
MME, 49, BF = 400.13 MHz, Solvent - CDCl3, 19 Oct 2020 T=298 K
1H NMR of 10e
NC
N
N
N
OMe
O
10e
8.03
8.02
8.02
8.01
7.96
7.95
7.93
7.93
7.91
7.90
7.66
7.65
7.64
7.64
7.63
7.62
7.62
7.61
7.61
7.59
7.59
7.57
7.57
7.56
7.51
7.51
7.49
7.49
7.26 Chloroform-d
5.20
3.78
2.00
2.07
5.34
2.12
2.00
3.00
f1 (мд)

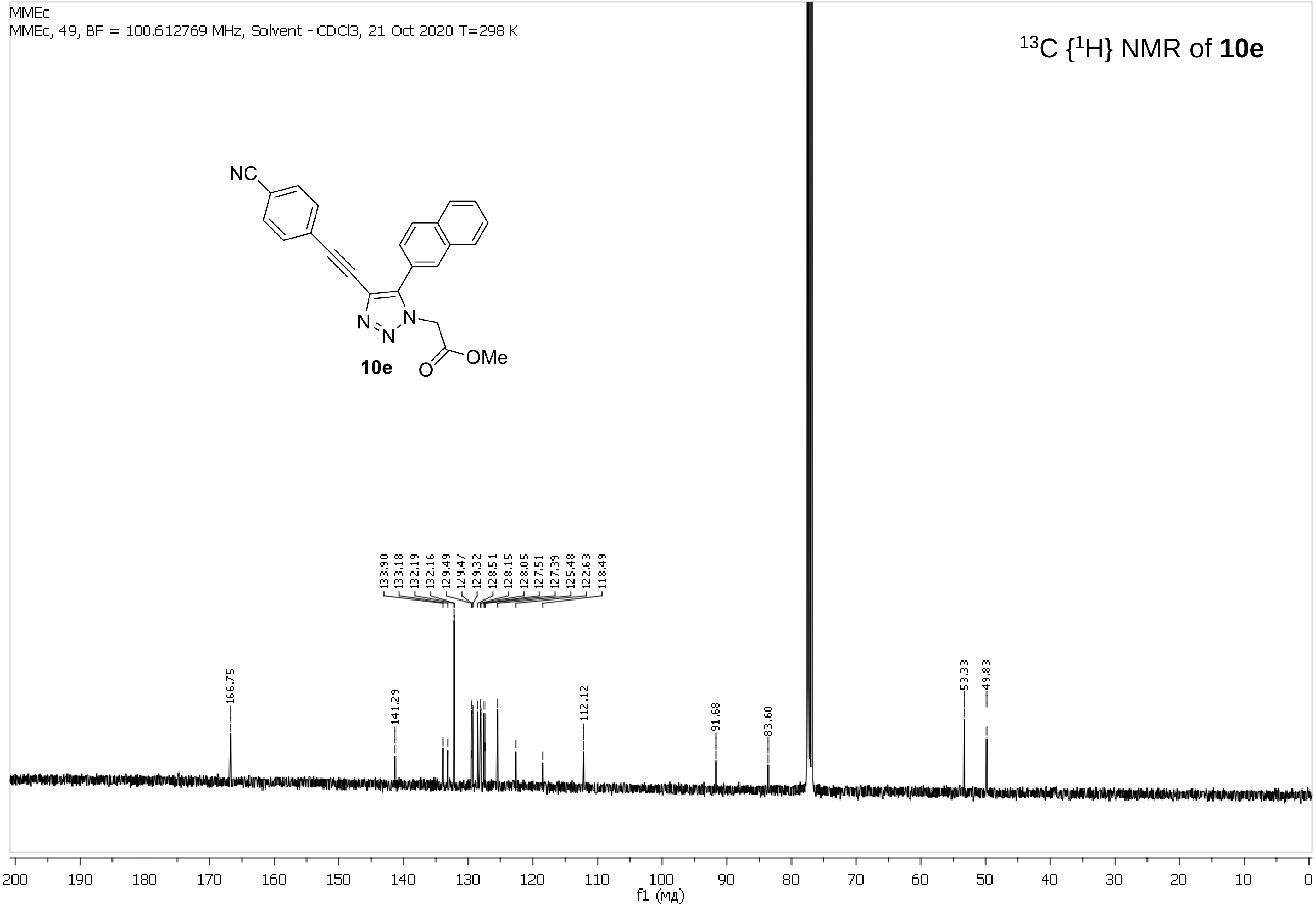
MMEc
MMEc, 49, BF = 100.612769 MHz, Solvent - CDCl3, 21 Oct 2020 T=298 K
^{13}C {^{1}H} NMR of **10e**
NC
N
N
N
OMe
O
10e
133.90
133.18
132.19
132.16
129.49
129.47
129.32
128.51
128.15
128.05
127.51
127.39
125.48
122.63
118.49
166.75
141.29
112.12
91.68
83.60
53.33
49.83
200 190 180 170 160 150 140 130 120 110 100 90 80 70 60 50 40 30 20 10 0
f1 (мд)

MMEd
MMEd, 49, BF = 100.612769 MHz, Solvent - CDCl3, 21 Oct 2020 T=298 K

^{13}C dept NMR of **10e**

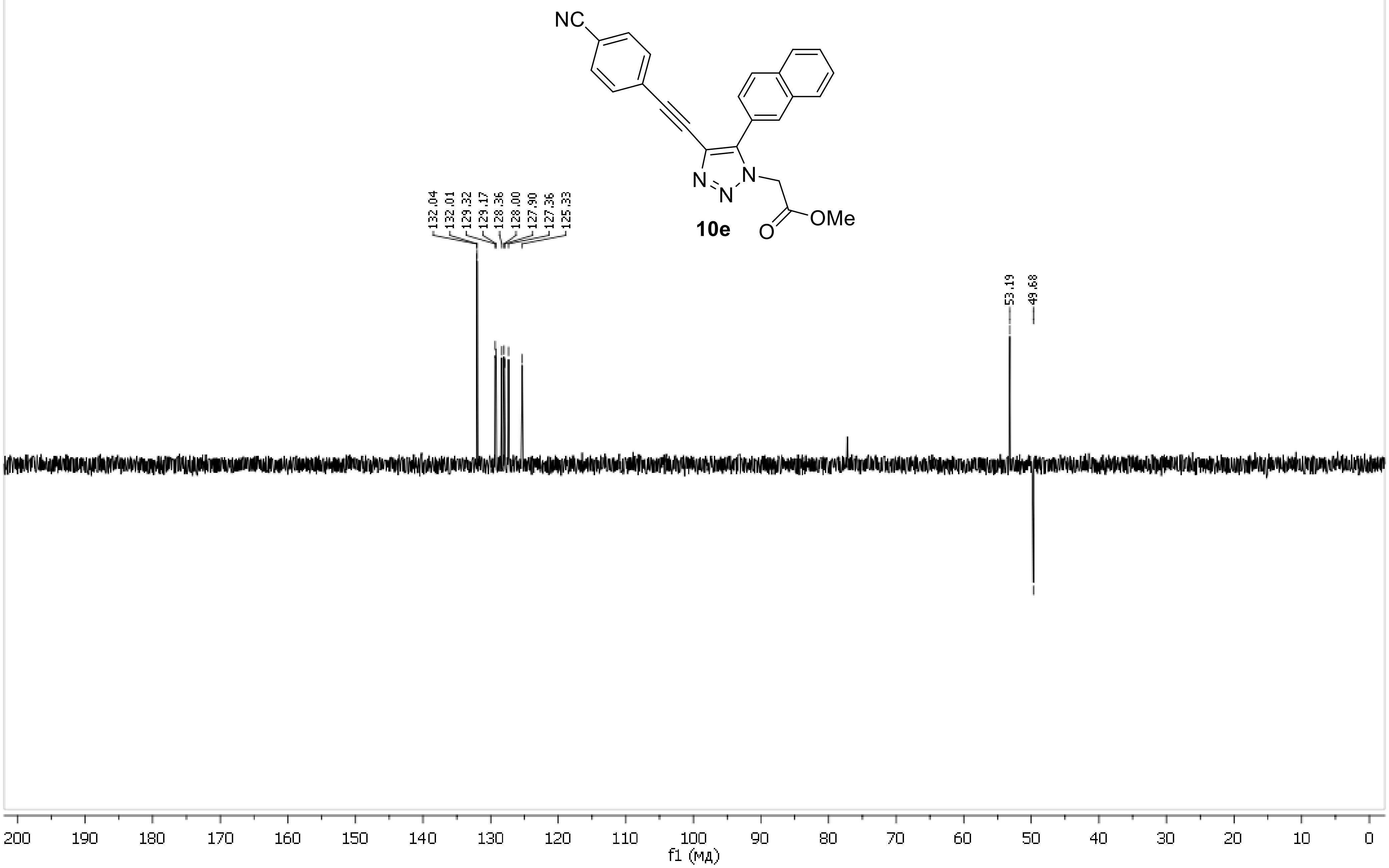

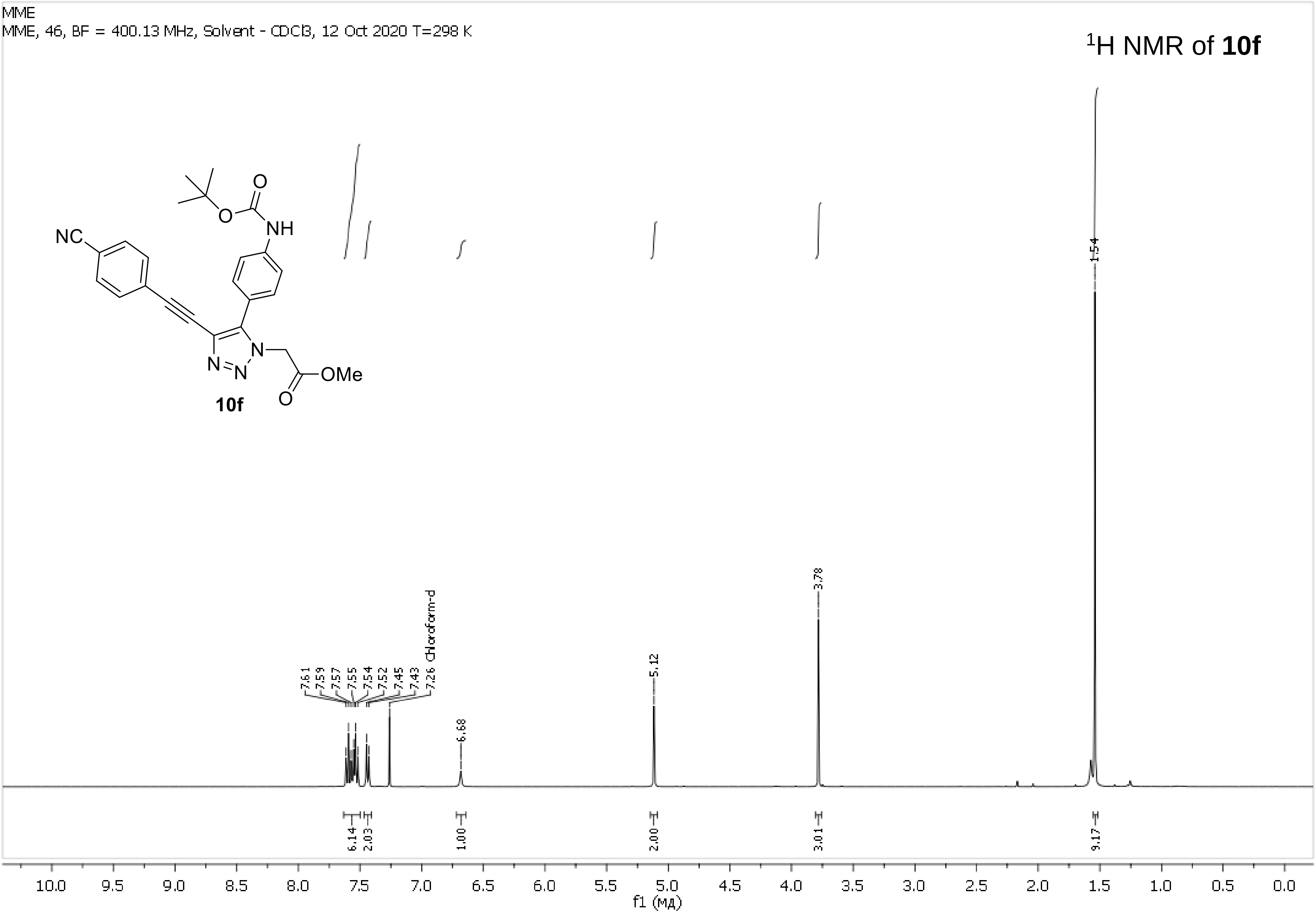
MME
MME, 46, BF = 400.13 MHz, Solvent - CDCl3, 12 Oct 2020 T=298 K
1H NMR of 10f
NC
O
NH
O
N
N
N
OMe
O
10f
7.61
7.59
7.57
7.55
7.54
7.52
7.45
7.43
7.26 Chloroform-d
6.68
5.12
3.78
1.54
6.14
2.03
1.00
2.00
3.01
9.17
10.0 9.5 9.0 8.5 8.0 7.5 7.0 6.5 6.0 5.5 5.0 4.5 4.0 3.5 3.0 2.5 2.0 1.5 1.0 0.5 0.0
f1 (мд)

MMEc
MMEc, 7, BF = 100.612769 MHz, Solvent - CDCl3, 14 Oct 2020 T=298 K

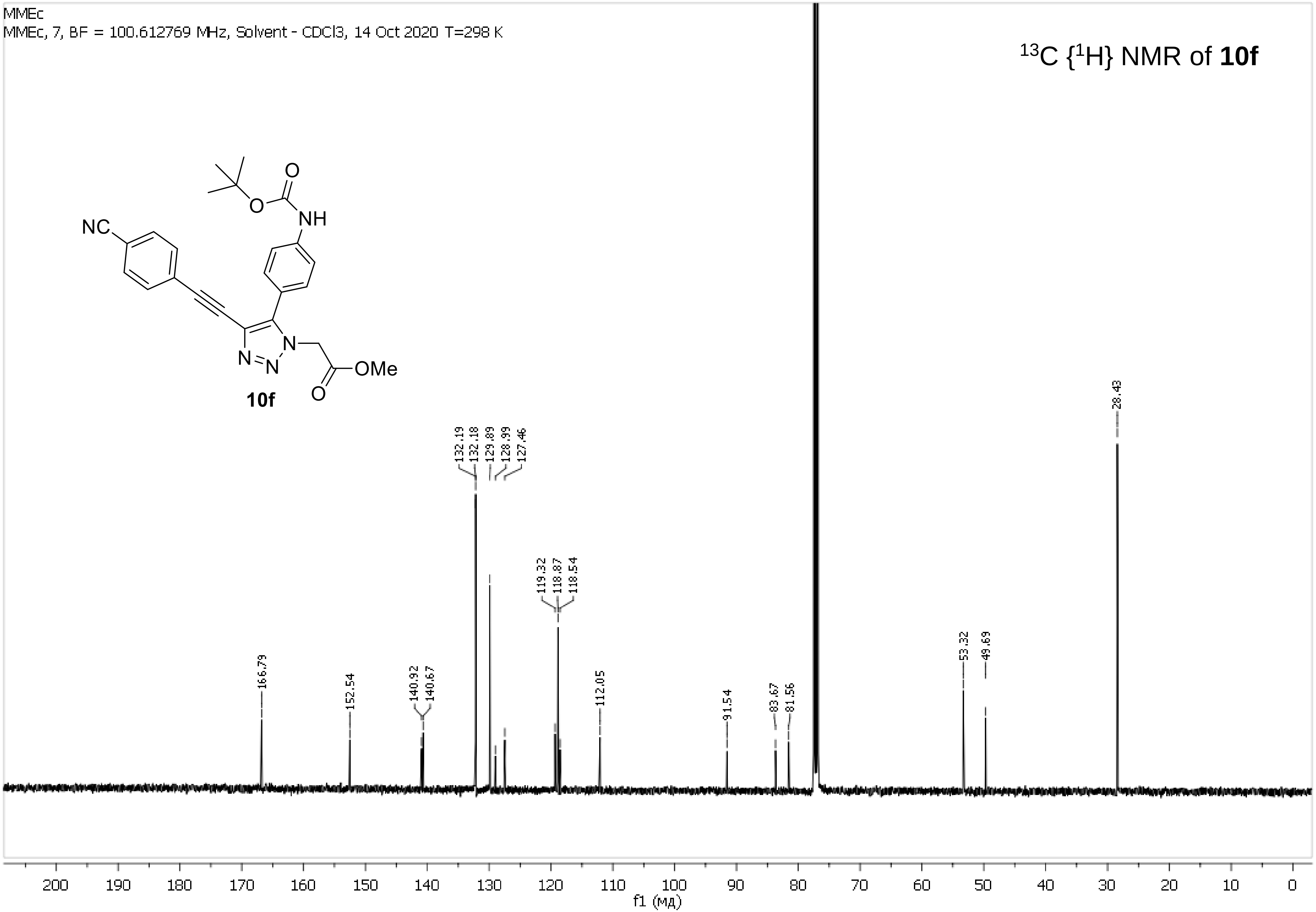

MMEd
MMEd, 7, BF = 100.612769 MHz, Solvent - CDCl3, 14 Oct 2020 T=298 K

^{13}C dept NMR of **10f**

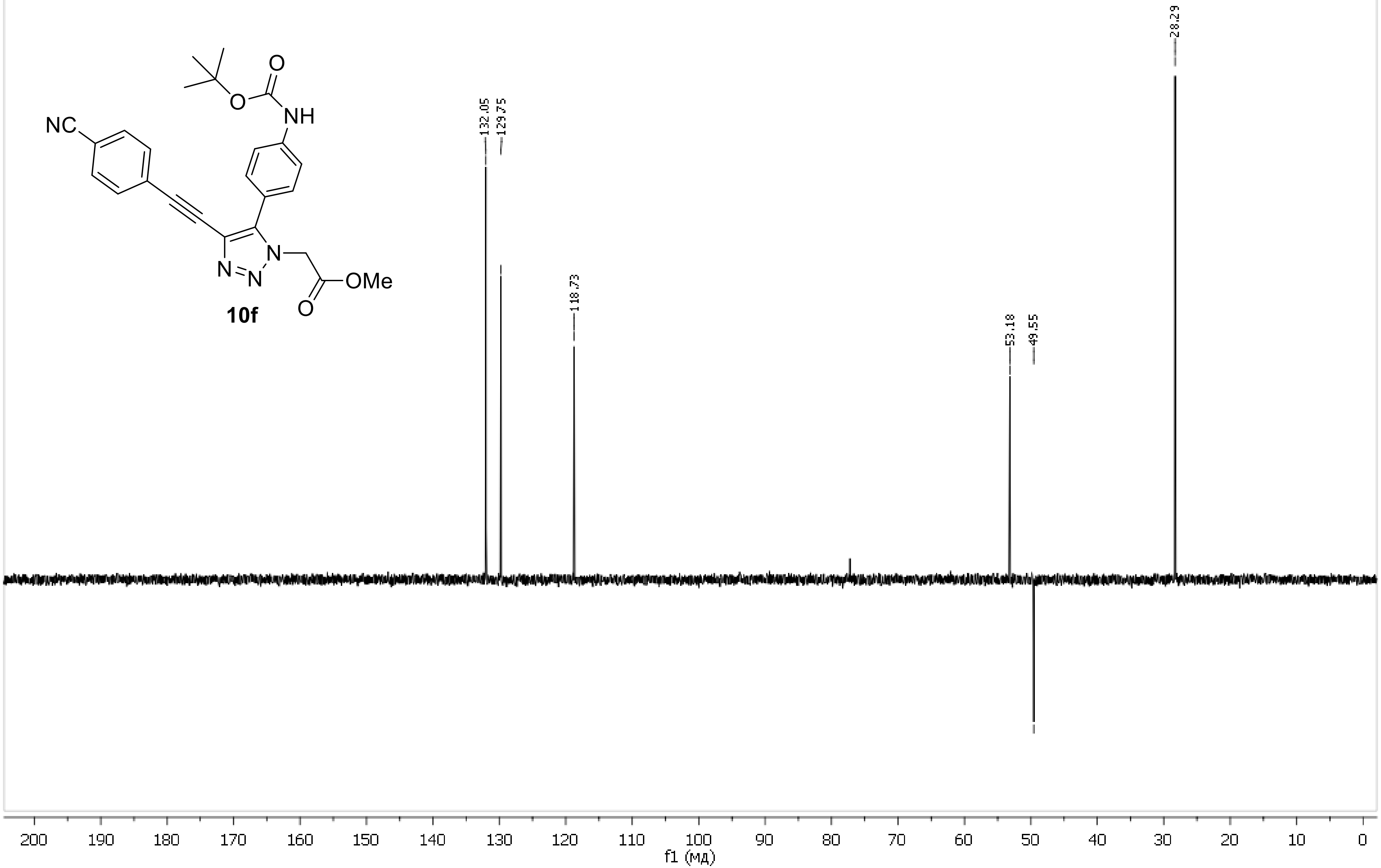

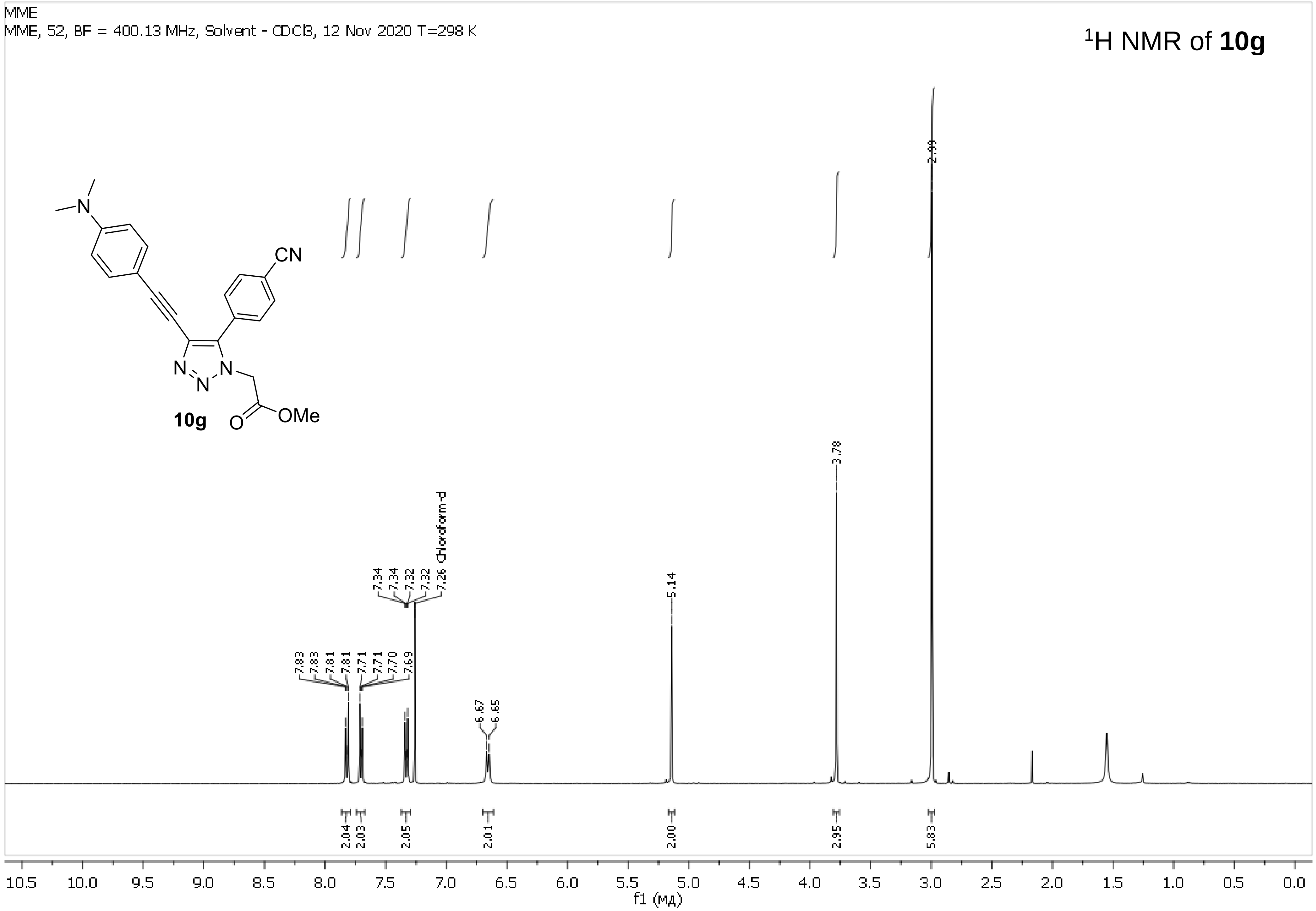
MME
MME, 52, BF = 400.13 MHz, Solvent - CDCl3, 12 Nov 2020 T=298 K
1H NMR of 10g
CN
N
N
N
N
OMe
O
10g
7.83
7.83
7.81
7.81
7.71
7.71
7.70
7.69
7.34
7.34
7.32
7.32
7.26 Chloroform-d
6.67
6.65
5.14
3.78
2.99
2.04
2.03
2.05
2.01
2.00
2.95
5.83
10.5 10.0 9.5 9.0 8.5 8.0 7.5 7.0 6.5 6.0 5.5 5.0 4.5 4.0 3.5 3.0 2.5 2.0 1.5 1.0 0.5 0.0
f1 (мд)

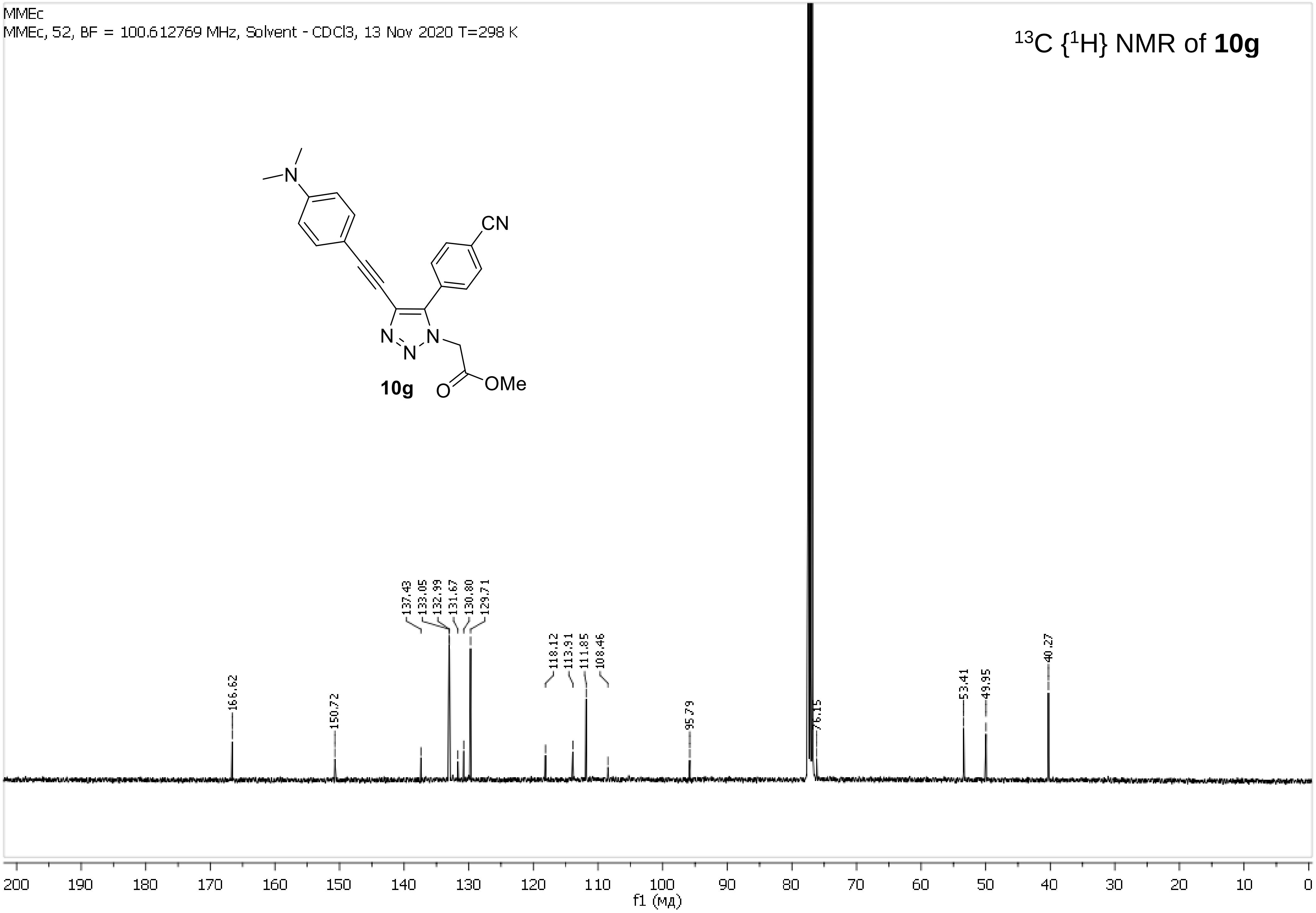
MMEc
MMEc, 52, BF = 100.612769 MHz, Solvent - CDCl3, 13 Nov 2020 T=298 K
13C {1H} NMR of 10g
N
CN
N
N
N
10g
O
OMe
166.62
150.72
137.43
133.05
132.99
131.67
130.80
129.71
118.12
113.91
111.85
108.46
95.79
76.15
53.41
49.95
40.27
200 190 180 170 160 150 140 130 120 110 100 90 80 70 60 50 40 30 20 10 0
f1 (мд)

MMEd
MMEd, 52, BF = 100.612769 MHz, Solvent - CDCl3, 13 Nov 2020 T=298 K

^{13}C dept NMR of **10g**

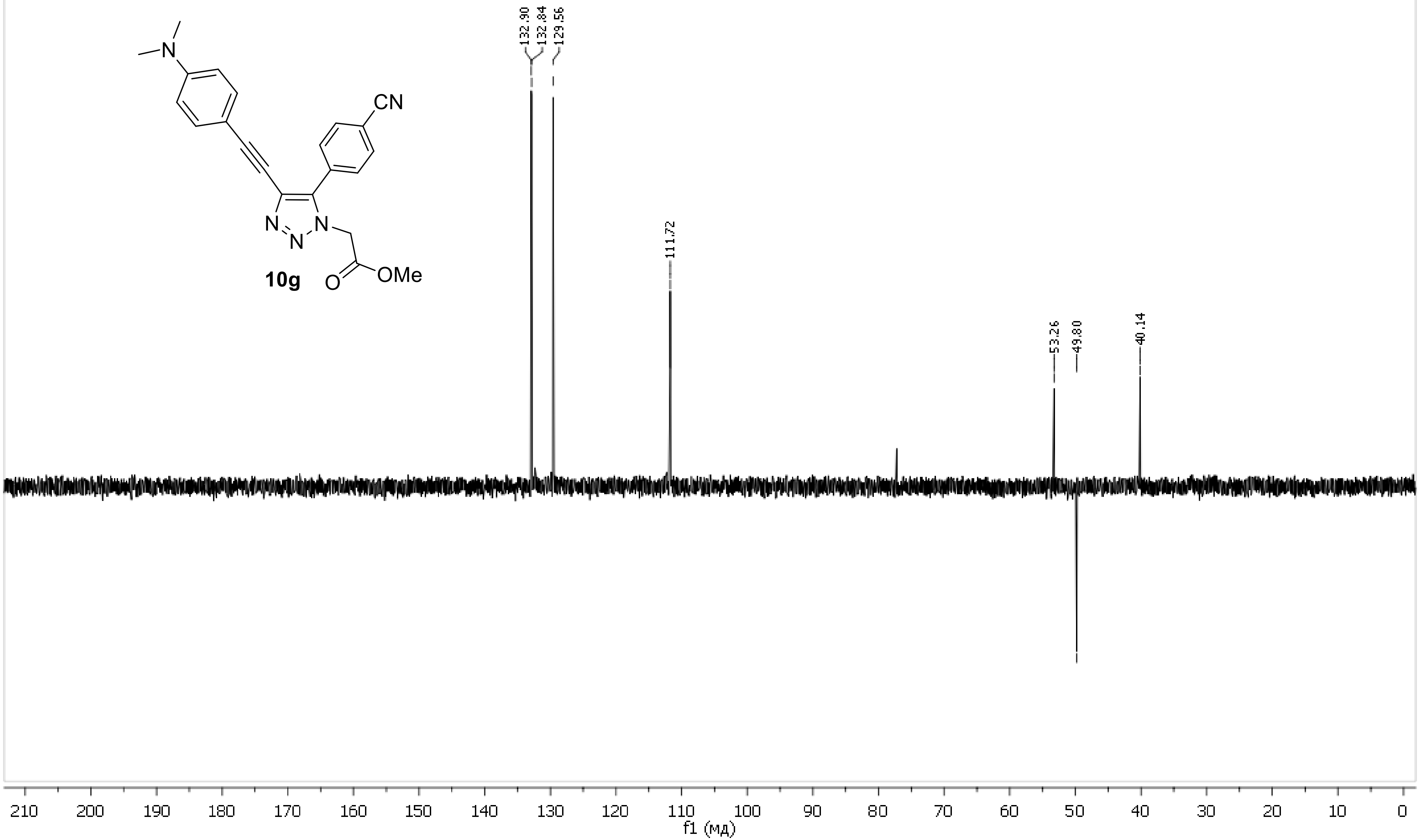

Supplement: Supplementary file 1 [file molecules-26-02801-s001.zip › molecules-1209038-supplementary.pdf]
